# Control of maleic acid-propylene diepoxide hydrogel for 3D printing application for flexible tissue engineering scaffold with high resolution by end capping and graft polymerization

**DOI:** 10.1186/s40824-022-00318-x

**Published:** 2022-12-09

**Authors:** Hao Nguyen Tran, In Gul Kim, Jong Heon Kim, Eun-Jae Chung, Insup Noh

**Affiliations:** 1 Department of Chemical and Biomolecular Engineering, Seoul National University of Science and Technology, Seoul, 01811 Republic of Korea; 2 Department of Otorhinolaryngology-Head and Neck Surgery, College of Medicine, Seoul National University Hospital, Seoul, 03080 Republic of Korea; 3 Convergence Institute of Biomedical Engineering and Biomaterials, Seoul National University of Science and Technology, Seoul, 01811 Republic of Korea

**Keywords:** 3D printing, Hydrogel ink, Scaffold, Poly(malate-*co*-propylene oxide) copolymer, Lipoic acid

## Abstract

**Background:**

Control of 3D printing of highly tough hydrogel inks with adequate printability, scaffold fidelity and mechanical properties are highly desirable for biomedical and tissue engineering applications. However, developing a biocompatible tough ink with high-resolution printability, biodegradability, self-healing, adhesion, and integration with surrounding tissues is a big challenge in 3D printing. The aim of this study was to develop extrusion-based 3D printing of viscous hydrogel composing of maleic acid and propylene diepoxide by controlling continuous mechanisms of condensation and radical polymerization.

**Methods:**

The molecular weight of highly adhesive propagating poly(malate-*co*-propylene oxide) copolymer was controlled by capping its growing chain with mono-functional lipoic acid with different compositions during condensation reaction to form lipoic acid capped gel (LP-capped gel). Poly(ethylene oxide)-diacrylate, PEGDA, is graft-polymerized to the LP-capped backbone polymer (MPLE gel) by UV irradiation during 3D printing process to control the properties of gel printability, mechanical properties, and cell adhesiveness and post-printing fidelity of the printed scaffolds with high resolution and mechanical properties (MPLE scaffold). The scaffolds in complex geometries have been printed out in diverse forms with addition of model drugs with different molecular weights and chemical structures. Both the highly adhesive LP-capped gel and printing-controlled MPLE gel/scaffolds are diversely characterized and compared with for their applications to the extrusion-based printability, including biocompatibility, self-healing, drug releasing, adhesiveness, multi-layered high-resolution printing. Further in vitro/in vivo tests were done to observe cytotoxicity, immune response and tissue formation by using different cells in mice model.

**Results:**

LP-capped hydrogel from maleic acid and propylene diepoxide gel showed control of gel properties with lipoic acid with one function group of thiol during condensation reaction, and the ratio at 1:0.3 (w/v) between LP-capped gel and PEGDA was chosen for the optimal results during radical polymerization process for 3D printing at high resolution (90-140 μm in strut thickness) with various complex geometries (lattice, rhombus, and honeycomb). The hydrogel showed excellent properties of self-healing, mechanical strength, biocompatibility, etc. In addition, the long-term release profiles of bioactive molecules were well-controlled by incorporating drugs of high molecular bovine serum albumin (BSA, 21 days, 98.4 ± 0.69%), or small molecule ornidazole (ORN, 14 days, 97.1 ± 1.98%) into the MPLE gel scaffolds for the tests of potential therapeutic applications. More importantly, the MPLE gels represents excellent in vitro cyto-compatibility against osteoblast-like cells (MC3T3) with viability value at 96.43% ± 7.48% over 7 culturing days. For in-vivo studies, the flexible MPLE scaffolds showed significant improvement on angiogenesis with minor inflammatory response after 4-week implantation in mice.

**Conclusion:**

The MPLE gel inks was well-controlled for the fabrication of flexible complex tissue engineering scaffold with high resolutions, shear-thinning, 3D printability and post-printing fidelity, by modulating the composition of the highly adhesive LP-capped gel and inert PEGDA as well as end capping of lipoic acid to the propagating poly(malate-*co*-propylene oxide) copolymer. The gel ink demonstrated its excellent printability, in vitro*/*in vivo biocompatibility and mechanical properties as well as sustained drug release from the gel.

**Graphical Abstract:**

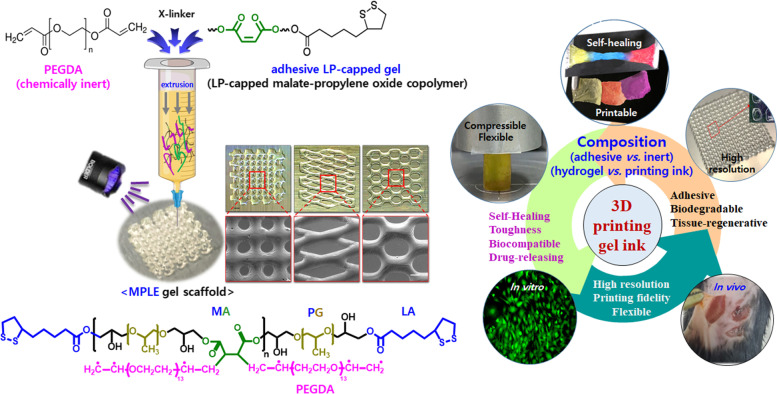

**Supplementary Information:**

The online version contains supplementary material available at 10.1186/s40824-022-00318-x.

## Introduction

Recently, extrusion-based 3D printing of adhesive hydrogels have attracted significant attention in the biomedical and tissue engineering fields owing to its exceptional capabilities in developing complex 3D structures, mostly to produce precisely designed scaffolds mimicking the native structures of patient tissues and organs [1–7]. Even though quite a few hydrogels have been reported for such extrusion-based 3D printing applications, still a suitable printable hydrogel ink with printing-related functionalities is the need of the hour. In specific, the ideal printing hydrogel ink requires the 3D printability and biomaterial functions such as high-resolution printing with fidelity, tunable degradation, controlled swelling, convenient sterilization, cell compatibility, adhesiveness, adjustable mechanical & rheological properties, drug or protein incorporation & release and post-printing fidelity [8–10]. Also, the shear-thinning property and adequate viscosity are important prerequisites for 3D fabrications using pneumatic-based extrusion 3D printing systems [5, 11, 12]. If the gel viscosity is too high for the printing process, then high air pressure maybe not possible to extrude the gels for printing [13]. On the other hand, the low viscosity and slow shear-thickening may restrain printing resolution and structural reproducibility of the original model during and after printing [14] Furthermore, the biocompatibility of the adhesive hydrogel inks is also significantly important for both in vitro and in vivo applications [4, 5, 13, 14].

Modulation of rheological and shear-thinning properties is essential for development of a 3D printable tough hydrogel with shape fidelity by using both natural and synthetic polymers [15–18]. For instance, Guo et al. reported a mechanically tough hydrogel with double networks synthesized by combining natural polymers of hyaluronic acid and alginate with excellent mechanical strength [19]. Langer group reported tough hydrogel in diverse forms with flexibility and adhesiveness by using maleic acid and propylene diepoxde through proliferating condensation polymerization. However, it has not been tried to be applied for 3D printing [20].

In this study, we report control of its hydrogel properties by adding lipoic acid with mono-acid group to the molecularly growing maleic acid-propylene diepoxide prepolymer under condensation polymerization. Further, for control of 3D printability we incorporate poly(ethylene glycol) diacrylate *via* radical polymerization, obtaining the poly(maleate-propylene oxide)-lipoate-poly(ethylene oxide) (MPLE) gel as a second step. The MPLE ink demonstrated high resolution 3D printing scaffold with fidelity by using four biocompatible components such as maleic acid and lipoic acid as natural biomaterials, and poly(ethylene oxide) and propylene oxide as synthetic ones. Maleic acid, a precursor to either fumaric acid or its parent maleic anhydride has emerged as promising candidate in tissue engineering applications by utilizing its advantageous biological properties such as excellent biocompatibility and strong redox activity for energy metabolism [21, 22]. Lipoic acid, dithiolane-3-pentanoic acid derived from organic compound, plays an important role in energy metabolism that can convert blood sugar in our body into energy by using oxygen through aerobic metabolism. Both poly(propylene glycol) [23–25] and poly(ethylene glycol) [26–28] have been engaged as important hydrophilic and biocompatible synthetic polymers in biomedical applications due to their excellent biocompatible properties such as biocompatible, drug-deliverable, non-immunogenic and ingestible.

As a first step, poly(malate-propylene oxide) was synthesized through a condensation polymerization *via* caffeine-catalyst, by using a ring opening reaction mechanism between maleic acid and propylene diepoxide. Since poly(malate-propylene oxide) was not stable due to its growth in molecular weight, it was stabilized by capping its live epoxide with mono-functional lipoic acid (LA) through the same condensation action in the same pot in this study, thus obtaining the LP-capped gel. The LP-capped gel showed excellent properties as a biomaterial such as biocompatibility, adhesiveness, self-healing, toughness and etc. Lastly, chemically and biologically inert, biocompatible PEGDA was graft-polymerized to the LP-capped co-polymer chain during 3D printing process, thus obtaining 3D printable gel ink with controls of adhesiveness, flexibility, 3D printability, biocompatibility, tunable degradation, swelling, mechanical and rheological properties (MPLE gel). In addition, the releasing profiles of protein model (bovine serum albumin) and antibiotic (ornidazole) from 3D printed MPLE gel scaffolds are studied based upon different crosslinking density, designed patterns, or loading method with desirable molecular weights that provides unique advantages in drug release sustainability. Loading and distribution of diverse drugs in the printed scaffolds would lead to better control of sustained delivery from the scaffolds and its applications to tissue engineering.

Both the composition of PEGDA and LP-capped gel and degree of radical polymerization affected printability, adhesiveness, fidelity, flexibility and mechanical properties of the MPLE gel ink. Since PEGDA is chemically inert and hydrophilic, the printing and fidelity properties of MPLE gel ink was balanced between the composition of adhesive LP-capped gel and chemically inert PEGDA, which affected printability, mechanical properties, flexibility and post-printing fidelity. The MPLE gel ink is 3D printable in diverse designs such as lattice, rhombus, and honeycomb, and the printed scaffold can be conveniently sterilized in high temperature/pressure autoclave operation. They showed also high resolution and post-printing fidelity with controlled swelling in phosphate buffer saline (PBS) solution. These physiochemical and biological properties (in vitro and in vivo*)* of the MPLE hydrogel as a printing ink and its printed structures demonstrated its excellent applicability in biomedical engineering, especially complex tissue engineering.

## Materials and methods

### Materials

Oligomeric propylene glycol diglycidyl ether (Mn = 380 g/mol), oligomeric poly(ethylene glycol)-diacrylate (PEGDA, Mw = 700 g/mol), ornidazole (ORN), caffeine, maleic acid (MA), and Irgacure 2959 (2-hydroxy-4′-(2-hydroxyethoxy)-2-methylpropiophenone) were purchased from Sigma Aldrich Chemical Co. (St. Louis, Missouri, USA). Lipoic acid (LA) was obtained from Tokyo Chemical Industry Co., Ltd. (Tokyo, Japan). Alpha minimum essential medium (α-MEM), 3-(4,5-dimethylthiazol-2-yl)-2,5-diphenyltetrazolium (MTT), neutral red, live & dead cytotoxicity kit (Invitrogen, Carlsbad, CA, USA) and bromodeoxyuridine (BrdU, Roche, Germany) were used as received for in vitro assay in this study. In vivo studies related materials and their brands: xylazine (Bayer, Korea), zoletil (Virbac, Korea), 3,3′-diaminobenzidine stain, (Vector Labs, USA). All other chemicals and materials were purchased from Sigma Aldrich, unless mentioned and were used without any purification.

### Syntheses of lipoic acid-capped gel (interchangeably, MPL gel) and MPLE gel

MPL gel (lipoic acid-capped gel) was synthesized at standard lab conditions as follows. Maleic acid (1.89 g) and caffeine (0.35 g) were loaded into a 60 mL glass vial consisting of a magnetic bar for stirring and mixing. After addition of oligomeric propylene glycol diglycidyl ether (6.84 g) into the mixture of maleic acid and caffeine, the total mixture was stirred at 360 rpm for 3 h in a standard digital glass oil bath (Lab Korea, Korea) at 70 °C. Condensation polymerization of the 3-component mixture solution was achieved at a time point with an intense increase in viscosity of the solution because of the caffeine-catalysis reaction. Then, lipoic acid (0.68 g) was added directly into the same polymerization reaction vessel, i.e., a pot. Later, the reaction was processed for 24 h at 90 °C under nitrogen gas atmosphere to obtain a LP-capped gel, interchangeably, MPL gel. Next, the LP-capped gel was purified in 20 mL acetone for 2 days, by replacing the acetone with fresh one daily. After drying the LP-capped gel in a ventilated conventional dry air oven at 55 °C for 24 h, different amounts (w/v) of PEGDA were added with 3% (w/w) Irgacure 2959 for graft polymerization of PEGDA to the LP-capped gel. The ratio referred to lipoic acid-capped gel: PEGDA (1:0.1, 1:0.2, and 1:0.3 w/v) (See Fig. S1).

### 3D printing and printability of the MPLE gels using extrusion-based 3D printer

Different infill structures of printing models (11 mm × 11 mm in lattice, rhombus and honeycomb) were designed in SolidWorks (Dassault Systems SolidWorks Corp, USA) and exported as STL files. The STL files were then imported into Simplify3D software (Simplify 3D version 4.0, Cincinnati, OH, USA) and converted into G-Codes. Then, the G-codes were modified and changed with codes acceptable for the Motion Master software version. The G-Code printer instructions were loaded in the computer connected to the 3D printer system as reported in our previous works to generate the layer-by-layer patterns for the input 3D design [29–32]. The printing process was optimized using our customized in-house built 3D printer with rotating extruder head by changing the different printing parameters like air pressure, printing speed, nozzle size, hydrogel composition, layer height, etc. [29–31, 33]. After optimizations, series of processing parameters including the layer height (250 μm), strut thickness (550 μm), and printing velocity (75 mm/min) were chosen for all printed structures. The prepared hydrogel was extruded after loading into a 5 mL syringe fitted with 27-gauge plastic needle (Musashi Engineering Inc., Japan). The 3 component mixtures of MPL intermediate, PEGDA and Irgacure 2959 were printed onto a glass coverslip under an air pressure of 225 kPa. Immediately after printing, the samples were crosslinked with UV irradiation using a UV trans-illuminator (UVT-2126, OPTIMA, USA) for 10 min and washed in PBS (pH 7.4) solution for three times to obtain the 3D structures of the MPLE scaffolds.

### Characterizations (SEM, FTIR and TGA)

The surface morphologies of the LP-capped gel fibres and 3D printed MPLE scaffolds were investigated using scanning electron microscope (SEM; Tescan Vega 3, Czech Republic) with an accelerating voltage of 15 kV. For the gel fibre test, the LP-capped gel was manually stretched into very thin wire/fibres, then plunged in a liquid nitrogen slush for 20 min. Afterwards, the frozen samples were freeze-dried in a lyophilizer at − 80 °C for 3 days. Similarly, the surface morphologies of the 3D printed MPLE scaffolds were observed under SEM after treatment with liquid nitrogen for 20 min and lyophilization for 3 days. All the dried samples were sputter coated with gold using standard methods before observation. Prior to the FTIR and TGA, the gel samples were frozen under − 80 °C for 2 days and subsequently, freeze dried at − 80 °C for 7 days to obtain dried samples. FTIR spectra of the dried MPLE gel and other samples were recorded and compared with those of the LP-capped gel samples using attenuated total reflectance-Fourier transform infrared spectroscopy (ATR-FTIR) spectrometer (Travel IR, Smiths Detection, USA) by setting the wavelength range from 500 to 4000 cm^− 1^ (Fig. S2). The thermogravimetric analyses (TGA, DTG-60, Shimadzu, Japan) was utilized to evaluate the thermal stability after graft-polymerization of PEGDA to LP-capped gel. Approximately 15 mg of each dried hydrogel (*n* = 1) was kept under nitrogen atmosphere with a heating rate of 5 °C/min in the temperature range of 25 °C to 650 °C.

### Rheology study

To analyse the addition of PEGDA effect on the viscosity of the LP-capped gel, rheology measurement was carried out on the MPLE gels and the LP-capped gel, with an R/S Plus Rheometer (Brookfield Engineering Labs; Middleboro, USA) at room temperature. Parallel plates with a diameter of 25 mm and the measurement gap set at 1 mm were used to determine the apparent viscosity of the hydrogel (2 ml) under varying shear rate. The shear rate was swept from 0.1 to 100 s^− 1^ and all the experiments were performed in triplicate for each sample.

### Texture profile analysis (TPA)

After initial stretchability evaluation of the hydrogel in films and fibres (Figs. S3 and S4), the mechanical properties of all the 3D printed MPLE scaffold samples were determined by a Stable Micro System (TA. XT plus texture analyser, Surrey, UK). The texture profile analysis (TPA) of the samples (11 × 11 × 3 mm^3^) was performed under compression in the distance mode with a maximum distance of 1.5 mm. TPA setting used for measuring the results were pre-test speed: 5 mm sec^− 1^, test speed: 1 mm sec^− 1^ and post-test speed: 5 mm sec^− 1^ [34]. The analyses were performed in triplicate and their average values were reported. For compressive strength investigation, three uniform cylindrical samples (10 mm × 10 mm) were employed for measuring the compressive strength with a constant strain of 80% at room temperature (∼26 °C). The rectangular 3D printed scaffold samples (1 mm × 5 mm × 25 mm) were utilized for tensile test with a 50 N loadcell, at a deformation rate of 12 mm/min in air at room temperature (∼26 °C). The tensile strain was calculated as the fractional change of sample’s length under tensile force, and the stress was obtained by dividing the force by the initial cross-sectional area of the 3D printed scaffold sample. The toughness (kJ/m^3^) of gel samples was determined by the area under stress-strain curves [35, 36].

### Lap shear adhesion test

The adhesive strength of MPLE gels were investigated by lap shear adhesion test using the texture analyser as described above. The MPLE gels (15 mm × 25 mm × 1 mm) were sandwiched between two rectangular substrates (25 mm × 76 mm) with different materials from the glass (Marienfeld, Germany), copper, wood, and porcine skins. Rubber (latex glove, Sigma-Aldrich) and polyethylene plastic were cut into sheets and fully covered the glass slide for comparison with others. During the measurement, the gel samples were kept in between two substrates and compressed with 100 g weight for 10 min. Subsequently, two adhered species were pulled until separation at a speed of 12 mm/min. Repeatability and reversibility of adhesion on the various substrates were also recorded.

### Swelling study

0.5 mL of the lyophilized MPLE gel was immersed separately in the 10 mm well plate containing 5 mL of PBS solutions (pH 7.4) at 37 °C. At regular intervals, their weights were measured after blotting their surface water by tissue paper. The experiments were conducted in triplicate for each condition, and their swelling ratios were compared with that of the LP-capped gel and 3D printed MPLE scaffolds (4 layers, honeycomb printed structures) The swelling percentages of the gel samples were calculated by the following equation [37–39]:

$$\textrm{Swelling}\ \left(\%\right)=\frac{{\textbf{W}}_{\boldsymbol{swollen}}-{\boldsymbol{W}}_{\boldsymbol{dry}}}{{\boldsymbol{W}}_{\boldsymbol{dry}}}\textrm{x}\ 100\%$$

where, W_swollen_ is the gel weight after swelling and W_dry_ is the dry gel weight.

### In vitro degradation assay

Long-term stability of the MPLE gel was evaluated by immersing 0.5 mL of each gel in PBS (pH 7.4) at 37 °C. The degradation profile was also obtained by measuring their % weight loss at the time points of day 1, day 7, day 14, and day 21 and compared to the day 0.

### Porosity analysis

The pore structures of the 3D printed MPLE scaffolds were evaluated in both wet (maximum swelling) and freeze-dried conditions (minimal swelling) by measuring the pore areas of the optical images from a microscope (Leica S8 APO, Leica Microsystems, Wetzlar, Germany) using ImageJ Software (version 1.52a, National Institutes of Health, USA). Porosities of the printed samples were investigated by analysing the whole surface area and the pore area of the printed scaffolds using the following equation:

$$\textrm{Porosity}\ \left(\%\right)=\frac{A_p}{A_t}\ \textrm{x}\ 100\ \left(\%\right)$$

where *A*_*p*_ is the pore area of the printed gel, and *A*_*t*_ is the total surface area of the construct.

### Integrity factor (Ir) analysis

Ir value was measured to examine the printing capability of the hydrogel ink on z-axis using the below equation [40, 41] The 3D printing process was conducted by setting up 10 layers and using 27G needle for each gel condition, and the scaffold d heights were calculated by the ImageJ software. The experiment was repeated triplicate for statistical analysis.

$$\textrm{Ir}=\frac{\textrm{Scaffold}\ \textrm{thickness}}{\textrm{Design}\ \textrm{thickness}}$$

### Pore factor (Pr) analysis

Pr value was calculated using the below equation [40, 42] to determine the printing fidelity of the gel scaffolds compared to the designed structure. By changing the needle sizes (27G, 30G, 32G), each gel condition was printed in 2, 4, and 6 layers. The gel scaffolds were then imaged by the microscope, followed by measuring the perimeter and area of pores by the ImageJ software.

$$\Pr=\frac{\mathrm L^2}{16\text{A}}$$

where A and L are the actual area and perimeter of the printed pore, respectively. An ideal hydrogel ink with excellent shape fidelity must present printed pores, which perform perfectly square structure (Pr = 1). Pr values were obtained from 8 pores of a lattice scaffold for each gel condition.

### In vitro drug loading and release study

#### Loading of bovine serum albumin (BSA) and ornidazole (ORN) in gel

A model protein BSA (0.08, 0.12, and 0.16 μmol) and a model antibiotic small molecular ornidazole (ORN, 18, 36, and 54 μmol) were separately loaded in the MPLE gel by following two different loading methods for comparison. The first method was absorption-based drug loading, which was performed on the gel surface by soaking the ORN antibiotic (3.953 mg, 7.906 mg and 11.860 mg) in 4 mL of PBS (pH 7.4) solution in separate 20 mL glass vials. Afterwards, the dried 3D printed MPLE scaffold samples (4 layers, honeycomb printed structures) were immersed in the above solutions and placed in an orbital shaker (Rotamax 120, Heidolph, Germany) for 24 h. The ORN-loaded MPLE scaffold was then transferred to a lyophilizer kept at − 70 °C for 3 days. The second loading method was direct incorporation of target drugs into the MPLE scaffolds. In this regard, the ORN and BSA were separately incorporated in the LP-capped gel, followed by mixing PEGDA (33.6 wt%) and Irgacure 2959 (3 wt.%) powders by a sonicator. Afterwards, the mixture was directly 3D printed and irradiated under UV light to obtain the drug-loaded MPLE scaffolds.

#### Drug release study

The releasing profiles of BSA and ORN from the 3D printed MPLE scaffolds were recorded at pH 7.4 and 37 °C. The UV–Vis spectrophotometer (BioMATE 3, Thermo Scientific, Madison, USA) was utilized to measure the release profile of BSA and ORN from the MPLE scaffolds. Specifically, the drug-loaded samples were soaked into 10 mL buffer solutions in the 25 mL Teflon bottles (Daihan Scientific, Korea). At defined time intervals, aliquots were pipetted out for measurement and fresh medium was added by replacing with fresh buffer solution. The BSA and ORN release (%) were calculated on the basis of standard graphs obtained from the protein and drugs solutions of known concentrations, and the experiments were conducted in triplicate (*n* = 3 per each condition).

### In vitro cytotoxicity assays

The cytotoxicity study for the MPLE gel was evaluated according to a previous protocol reported from our labs [43–45]. Briefly, the round disk-shaped gel samples (500 μl, diameter = 10 mm) were prepared by spreading the gel on a glass coverslip and then rinsing it in a petri dish containing 10 ml of distilled water for 5 days by replacing the water daily. Afterwards, the hydrogel disk samples on the cover glass were autoclaved at 121 °C for 20 min. Subsequently, the sterilized samples were incubated in a 24-well plate after loading α-MEM media (10% fetal bovine serum and 1% penicillin-streptomycin) for 3 days. Three different kinds of in vitro cytotoxicity assays of MTT, Neutral Red, and BrdU were used to determine the cyto-toxicity of the MPLE gel against micro-organs such as mitochondria, lysosome, and DNA respectively [46, 47]. According to these assays, the extract solutions from the autoclaved Teflon sheet (negative control, diameter = 10 mm), latex layer (positive control, diameter = 10 mm), and the prepared MPLE gel disks for 3 days were utilized for the in vitro analysis. On the other hand, mouse osteoblast-like bone cells (MC3T3, Sigma Aldrich) with the density at 1× 10^4^ cells per well were in-vitro cultured in a 96-well plate and placed in an incubator with 5% CO_2_ at 37 °C for 24 h. Next, medium was removed and 100 μL of each above-mentioned extract solutions were added into each well and the absorption values were obtained using microplate reader [43, 44, 48]. The cell seeded MPLE gel disk samples were stained with Live and Dead stains as per the reported protocols. The live and dead images were captured at regular time intervals using a fluorescence microscope (Leica DMLB, Germany) (Fig. S5).

### In vivo biocompatibility analysis: histology and immunohistochemistry (IHC)

For in vivo investigation, 8 weeks old male C57bl/6 mice (Orient Bio Inc., Sungnam, Korea) were employed for animal test of 3D printed MPLE gel honeycomb scaffolds. Combination of Xylazine (Rompun®; Bayer, Monheim, Germany), and Zoletil (Virbac, Carros, France) were injected intramuscularly as anesthetizing agents with the dose concentration of 10 mg/kg and 20 mg/kg, respectively. After incising the dorsal skin, the MPLE honeycomb scaffolds were implanted into the subcutaneous layer. Post-implantation, the test subjects were overdosed with the anaesthesia and sacrificed at the time period of 1- and 4-weeks). From the implanted sites, skin tissue flaps were collected carefully for further investigation. Approved animal protocols from The Institutional Animal Care and Use Committee (IACUC) of the Seoul National University Hospital (No. 19–0026-S1A0) were implemented in this study.

The collected tissue samples were treated first with 4% paraformaldehyde for 24 h, dehydrated, and then embedded in paraffin wax before cutting in 5 mm thick sections (*n* = 5 per group). Haematoxylin/Eosin (H & E) and Masson’s trichrome (MT) staining procedures were carried out for the explanted samples, and then observed under a microscope and images. Immuno-histochemical analyses were performed as per our previous studies [30]. Briefly, the tissue samples were incubated with F4/80 (1:500; Abcam; Dawin Bio, Korea) and CD31 (1:300; Abcam, Dawin Bio, Korea) primary antibody stains at 4 °C overnight. Then, the samples were treated for 1 h at room temperature with horseradish peroxidase (HRP) coupled secondary antibodies (1:500). Finally, the samples were stained with 3,3′-diaminobenzidine stain (DAB Peroxidase Substrate) as per the previous protocol [30]. The prepared samples were observed under a light microscope (Olympus, Japan) and images were captured. The captured images were analysed using ImageJ software and CD31 & F4/80 positive blood vessels (CD31+ ducts per mm^2^) and macrophages (F4/80+ cells under × 40 high-power field) were quantified, respectively.

### Statistical analysis

All experimental data were presented as mean ± standard deviation (S.D.) and compared statistically using ONE-WAY ANOVA with Tukey’s multiple comparison tests in OriginPro 9 software. Differences were statistically significant when *p* ≤ 0.05 (*) and *p* ≤ 0.01 (**).

## Results

### Characterization of the developed hydrogel

**Fig. 1:**
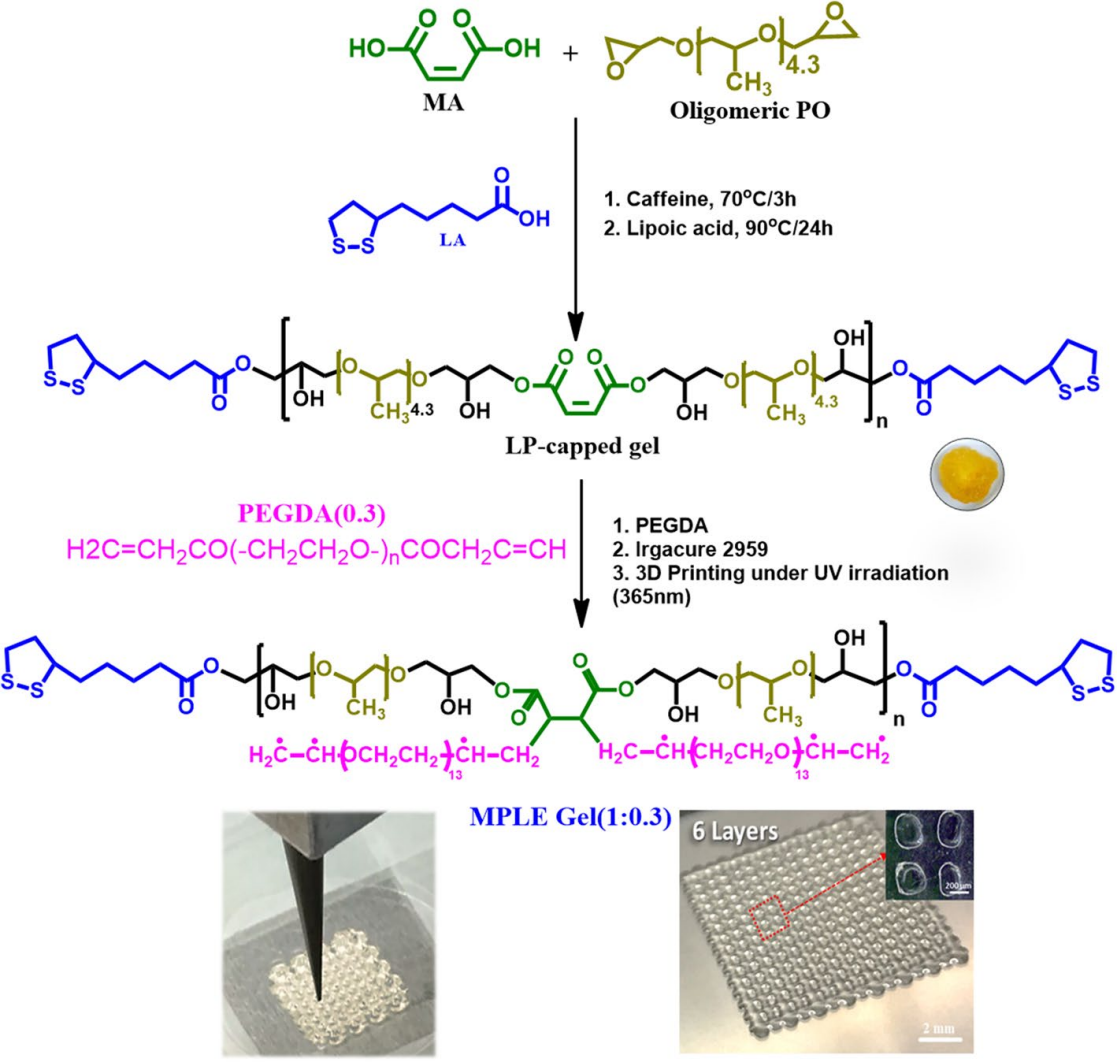
**A** Probable chemical schematic of the MPLE printing gel synthesis by the condensation polymerization of maleic acid, propylene oxide diacrylate and lipoic acid in a pot (i.e. the LP-capping gel) and then radical graft-polymerization of PEGDA during 3D printing. The 1(the LP-capped gel):0.3(PEGDA) ratio was chosen for the optimal results for 3D printing in this study

The formation of the LP-capped gel was readily achieved by adding up the mixture of oligomeric propylene oxide and maleic acid in a pot, thus capping the end groups of MP intermediate with lipoic acid. Caffeine has worked as a mild base to catalysis to build polymer networks, where in the presence of heat, caffeine abstracted protons from the acidic groups of maleic acid, allowing the formation of maleic acid, thus forming nucleophilic carboxylate of maleic acid in its both ends. The maleic carboxylates then started reacting with epoxide groups of propylene oxide and initiated the ring-opening of diepoxide from the oligomeric propylene oxide through caffeine catalyst. At this moment, the viscosity of the solution gradually increased, which implied the condensation polymerization between maleic acid and propylene oxide. Afterwards, lipoic acid was added into the MA gel solution, and then caffeine continued deprotonating carboxylic acid groups from LA to induce ring-opening of diepoxide. It is presumed that all reactive sites of excess lipoic acid were grafted with the epoxide end sites of propylene oxide to form a co-polymeric network. After forming maleic acid-propylene oxide (MP) copolymer, the end groups of the generated polymer chain are expected to be capped by lipoic acid since it has one acid in the end and a five ring at the other end, thus forming maleic acid-propylene oxide copolymer with lipoic acid end group, i.e. the LP-capped gel, which is highly adhesive. Finally, the predefined amounts of PEGDA were added to enhance the printability. We hypothesized that the PEGDA oligomers react with the unsaturated groups of maleic acid in the LP-capped copolymer network and initiated the radical polymerization during 3D printing. As suggested in Fig. 1, the ratios of 1:0.1, 1:0.2 and 1:0.3 (w/v) were employed for the MPLE gel synthesis and 3D printing, referred as MPLE gel (1:0.1), MPLE gel (1:0.2), and MPLE (1:0.3). It is showed that MPLE gel (1:0.3) was best fit to the 3D printing in this study. For the MPLE gel, the peak at 1642 cm^− 1^ was significantly reduced in terms of its intensity which indicated the occurrence of graft radical polymerization of PEGDA to the LP-capped co-polymer. Noticeably, there is a characteristic peak of C-S bonds near 676 cm^− 1^ appearing in both the MPLE and LP-capped polymers which confirm the ring-opening of di-epoxide groups and graft polymerization of PEGDA to the LP-capped co-polymer.

**Fig. 2:**
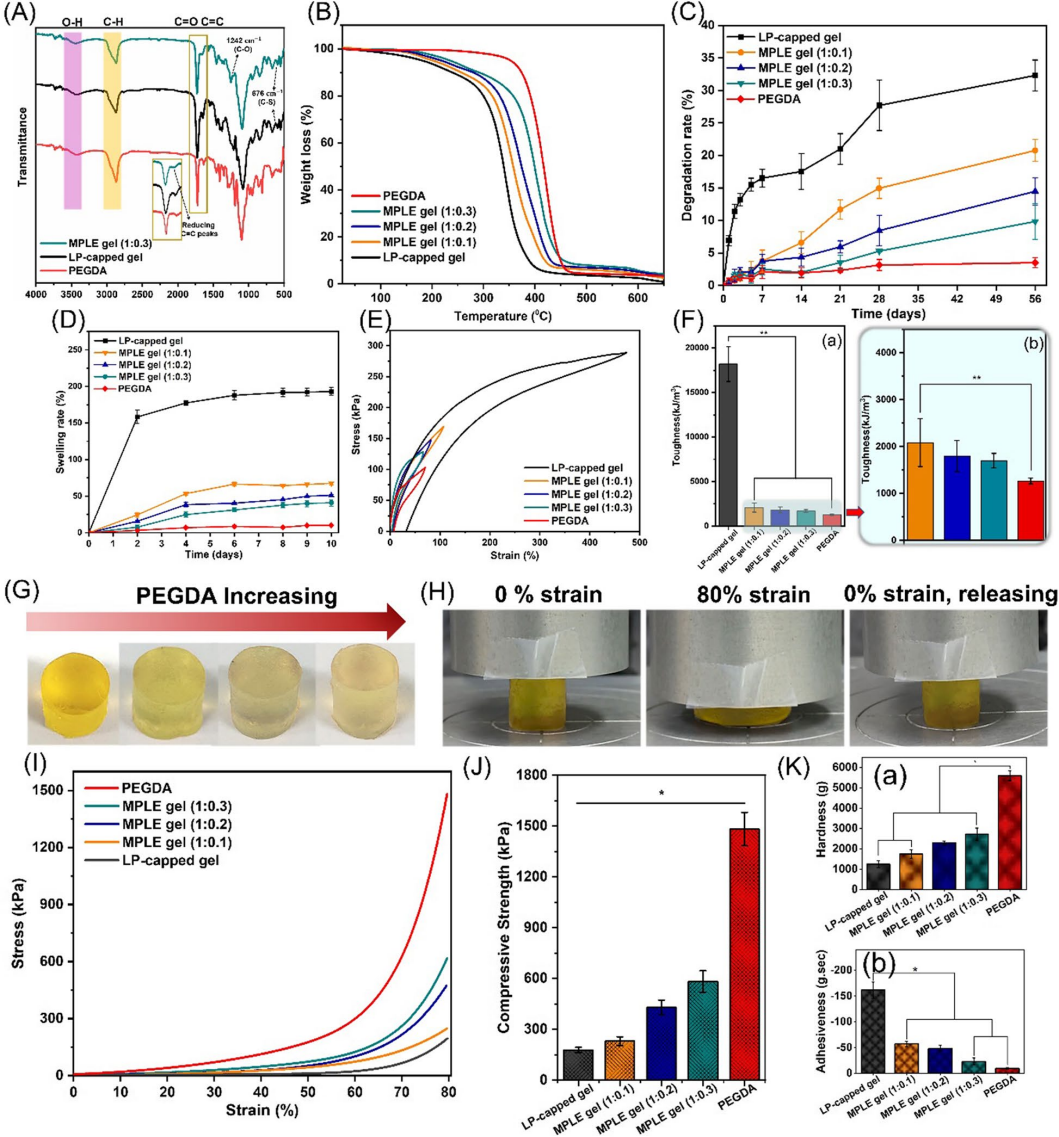
Analysis of the LP-capped and MPLE samples. **A** FTIR spectrums of MPLE samples and their monomeric compounds. **B** TGA, (**C**) in vitro degradation rate up to 56 days, (**D**) swelling behaviour, (**E**) tensile stress strain curves, (F-a) toughness of PEGDA, LP-capped and MPLE gel samples and (F-b) enlarged view of toughness of the gel samples. **G** Properties of the MPLE gels in uniform cylindrical shapes with color changes depending on the amounts of PEGDA in the MPLE gel; **H** the recovery of the MPLE gel (1:0.3) under compression mode (Initial state, 80% compression state and compression released); for PEGDA, LP-capped and MPLE gels, (**I**) Compressive stress-strain curves up to 80% strain; **J** their ultimate compressive strength and (**K**) Texture profile analysis (TPA), where (a) hardness and (b) adhesiveness values

FTIR spectra of all the samples of MPL, MPLE and PEGDA were shown in Fig. 2A. The peaks of the LP-capped samples at 3450 cm^− 1^, 2873 cm^− 1^, 1731 cm^− 1^ and 1642 cm^− 1^ were due to the O-H, C-H, C-H_3_, C=C stretching vibrations, respectively. The PEGDA spectra presented the characteristic peaks at 3450 cm^− 1^ (O-H bond), 2873 cm^− 1^ (C–H stretching), 1731 cm^− 1^ (C=O stretching), 1632 cm^− 1^ (C=C bond), and 1242 cm^− 1^ (C–O stretching) [44, 49, 50]. For the MPLE samples, the peak at 1642 cm^− 1^ was significantly reduced in terms of its intensity which indicated the occurrence of graft polymerization of PEGDA to the LP-capped copolymers. Noticeably, there is a characteristic peak of C-S bonds near 676 cm^− 1^ appearing in both the MPLE and LP-capped samples which confirm the ring-opening of di-epoxide groups and graft polymerization of PEGDA to the LP-capped copolymers.

Figure 2B shows the thermal stability and the weight change of the MPLE and the LP-capped samples over the temperature ranging from 25 °C to 700 °C in the TGA analysis, where three major weight loss zones can be observed. The first weight loss zone (between 130 and 170 °C) was attributed to the breakage of the maleic units [51]. The second weight loss region (between 170 and 320 °C) was owing to the disruption of lipoic acid chains [52] and the last one (between 330 and 420 °C) signifies the breakdown of the crosslinked polymer network from PEGDA [53, 54]. As shown in the graph (Fig. 2B), it is noticeable that the higher concentration of PEGDA in the MPLE samples resulted in smaller weight loss. The results also imply that addition of PEGDA to the LP-capped polymer network can improve the thermal stability of the MPLE samples due to its higher crosslinking density and potentially interpenetration by PEGDA graft polymerization to the previously formed co-polymer network [55]. Additionally, the weights of both the LP-capped and MPLE samples in 3D printing are stable up to 130 °C, which is very important to facilitate the sterilization method by high temperature/pressure autoclaving, which usually is set at 121 °C.

In vitro degradation rate of the MPLE samples was studied under standard in vitro conditions as mentioned in the method section (Fig. 2C). Among the tested samples, the degradation rate of PEGDA gel remained lowest with only 3.49 ± 0.76% at day 56. The LP-capped gel showed faster degradation rate over 28 days than the MPLE gels. After this period, the degradation trend was similar to all the tested gels. However, the percentage of degradation was 32.3.7 ± 2.35% for the LP-capped gel, whereas 14.96 ± 1.55%, 8.43 ± 2.33%, and 9.84 ± 2.73% for the MPLE gel (1:0.1), MPLE gel (1:0.2) and MPLE gel (1:0.3) samples, respectively at day 56. This fast degradation rate in the LP-capped gels perhaps attributed to the breaking down of the ionic bonds between the –OH and carbonyl groups of the co-polymeric macromolecules as well as the formation of its loose gel network. The slower degradation rate trend seen in the MPLE gels were due to the presence of the higher density of the crosslinking in 2D and 3D networks by the PEGDA themselves and chemical bonding between the unsaturated structures in the maleic acid and the acrylate groups of the PEGDA by the UV irradiation. As the PEGDA concentration increased, the crosslinking was higher, resulting in the cross-linked network with higher effective cross-linking density, forming lower possibility of hydrolysis of the MPLE polymer network. This kind of tuneable and controllable degradation rate and effective crosslinking density of the 3D printing hydrogel are highly applicable in biomedical fields. As per the requirement of the tissue or organ, the degradation rate can be adjusted by changing the ratio of the monomers and their chemical structures.

Swelling rate of the MPLE gel also showed similar trend to that of its in vitro degradation rate (Fig. 2D). The LP-capped gel showed 193 ± 5.42% swelling percentage after 6 days. However, after this period, the swelling graph of the gels acquired equilibration and reached plateau till 10 days. In case of the MPLE gel (1:0.1), MPLE gel (1:0.2) and MPLE gel (1:0.3) samples, the LP-capped (1):PEGDA(0.3), PEGDA(0.2) and PEGDA(0.1) gels, the swelling rate was much lesser than that of the LP-capped gel and the PEGDA gel. As the PEGDA ratio increased in the MPLE gels, the swelling rate decreased coinciding with the results of in vitro degradation studies. In this case, the UV irradiation crosslinking increased the formation of highly crosslinked dense networks in the MPLE gels. Even with a small addition of the PEGDA molecules, the MPLE gel’s swelling was largely reduced when compared to the LP-capped gel, which was lower than ~ 55% for all the MPLE gel samples. This kind of controlled gel swelling is highly applicable in case of 3D printing applications as they require lesser swelling rate in order to keep the 3D post-printing shape fidelity and their printing resolutions under moist conditions, i.e., in vitro and in vivo environments.

These results were supported by the analyses of the stress-strain curve and toughness of the gel samples. The highest stress-strain value of 288.83 ± 14.77 kPa at 473.36% was observed in the LP-capped gel while the PEGDA gel represented the lowest value of 103.057 ± 4.39 kPa at 70.06%, and as the PEGDA increased its value decreased to 169.49 ± 3.39 kPa (MPLE gel (1:0.1)), 147.97 ± 0.94 kPa (MPLE gel (1:0.2)), and 122.237 ± 5.70 kPa (MPLE gel (1:0.3)) **(**Fig. 2E). Their toughness followed these trends (Fig. 2F-a), i.e. the LP-capped gel showed the highest toughness at 18206.48 ± 1966.67 kJ m^− 3^ and then the PEGDA graft-polymerized gels showed significantly lower toughness values at 2085.69 ± 51.66 kJ m^− 3^, 1801.16 ± 331.23 kJ m^− 3^, and 1702.08 ± 155.14 kJ m^− 3^ for MPLE gel (1:0.1), MPLE gel (1:0.2), and MPLE gel (1:0.3), respectively (Fig. 2F-b). As envisioned, the graft polymerization of the PEGDA to the LP-capped polymer network showed possibilities of control of the extrusion-based 3D printability by modulating the amount of PEGDA, the degree of swelling, toughness and stress-strain phenomena.

Figure 2G shows the LP-capped gels and MPLE gels obtained by graft-polymerization of the LP-capped gel with three different amounts of PEGDA. As the PEGDA contents increased, the colour of the MPLE gel faded away from dark yellow to light yellowish colour. These gel samples with uniform cylindrical shapes were used for their mechanical testing. In Fig. 2H, the MPLE gel (1:0.3) at initial state (0% strain), followed by compressed state (80% strain) and then, recovery state (removal of compression strain to 0%) were observed with digital images. Even at 80% strain, the gels did not break or disintegrate. Also, when the strain was removed, the LP-capped and MPLE gels retained its shape immediately without noticeable hysteresis. There was no visible loss of shape or matrix during and after the compression test. The recovery of the compressed gels improved according to the amount of the PEGDA added to the LP-capped gel (Fig. 2I). As the ratio of PEGDA increased from 0.1, 0.2 and 0.3, the gels become stronger and more flexible due to higher effective crosslinking density of the PEGDA network, which has been graft-polymerized from the unsaturation of maleic acid in the repeating unit of LP-capped copolymer networks. This trend can be evidently seen from the graphs (Fig. 2I-J). As observed in Fig. 2J, the MPLE (1:0.3) gels were ~ 300% stronger than the control LP-capped gel. Likewise, the other MPLE samples also exhibited higher compressive strength than the LP-capped gel. As the ratio of PEGDA increased in the LP-capped gel, the compressive strength increased significantly. Similarly, in the texture profile analysis (TPA), the addition of PEGDA in the MPLE gels showed improved other mechanical properties compared to the control LP-capped gel. For instance, hardness values of the MPLE gel (1:0.3) was ~ 225% higher than the LP-capped gel (Fig. 2K-a). However, the adhesiveness value of the LP-capped gel was higher than the MPLE samples due to increase in chemical inertness of the PEGDA polymer structure in the MPLE gel networks (Fig. 2K-b). The modulation of the gel ink’s adhesiveness properties by the ratios of lipoic acid and PEGDA can help its printability in the extrusion-based printing process as the gel’s printability could be controlled by modulating its fluidic dynamics through the nozzles. So, the addition of PEGDA led to the reduction of the adhesiveness, toughness and viscosity as described below of the MPLE gels, but the increase in their elasticity, flexibility and compression properties, affecting on their printability. These experimental results are reassuring the importance of the required properties in the extrusion-based 3D printing, where the gel ink requires control of healing ability, moderate adhesiveness, shear-thinning ability with tuneable viscosity, controlled mechanical properties with improved compressive and elastic recovery from repeated hysteresis.

### Adhesion test

**Fig. 3:**
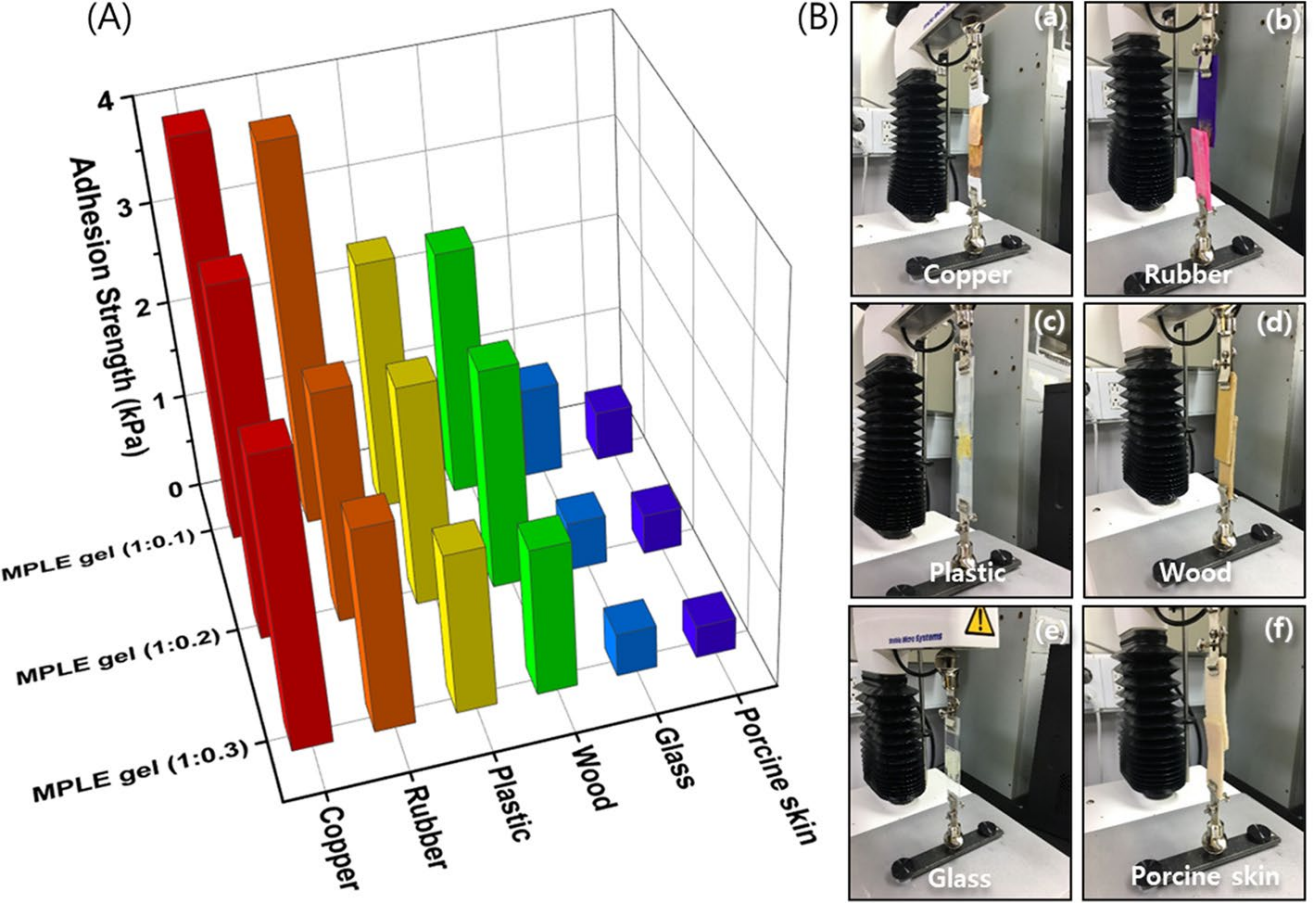
**A** Adhesion strength of the MPLE gels on different surfaces. **B** Digital images of adhesion test on copper(a), rubber(b), polystyrene plastic(c), wood(d), glass(e), porcine skin(f)

To understand gel ink’s flow through the 3D printing nozzle, the adhesive property of the MPLE gels was qualitatively examined through the lap shear test by placing the gel between two rectangular substrates, which derived from both inorganic and organic surfaces. As shown in the Fig. 3A the adhesion strength of the MPLE gel (1:0.1) with lowest amount of PEGDA on the copper surface was highest (4.04 kPa), compared to other surfaces, probably due to higher metal ionic interactions. The hydrogel also represented moderate adhesion to rubber (3.86 kPa), followed by polystyrene plastic (2.56 kPa), wood (2.51 kPa), glass (0.93 kPa) and porcine skin (0.52 kPa). Notably, MPLE gels with an increasing addition of PEGA represented a decrease in the adhesion properties on copper (3.53 kPa and 2.96 kPa), rubber (2.37 kPa and 2.09 kPa), polystyrene plastic (2.25 kPa and 1.65 kPa), wood (2.25 kPa and 1.51 kPa), glass (0.51 kPa and 0.44 kPa), and porcine skin (0.41 kPa and 0.33 kPa) for MPLE gel (1:0.2) and MPLE gel (1:0.3), respectively.

### Rheological analysis and characterization of the 3D printed hydrogels

**Fig. 4:**
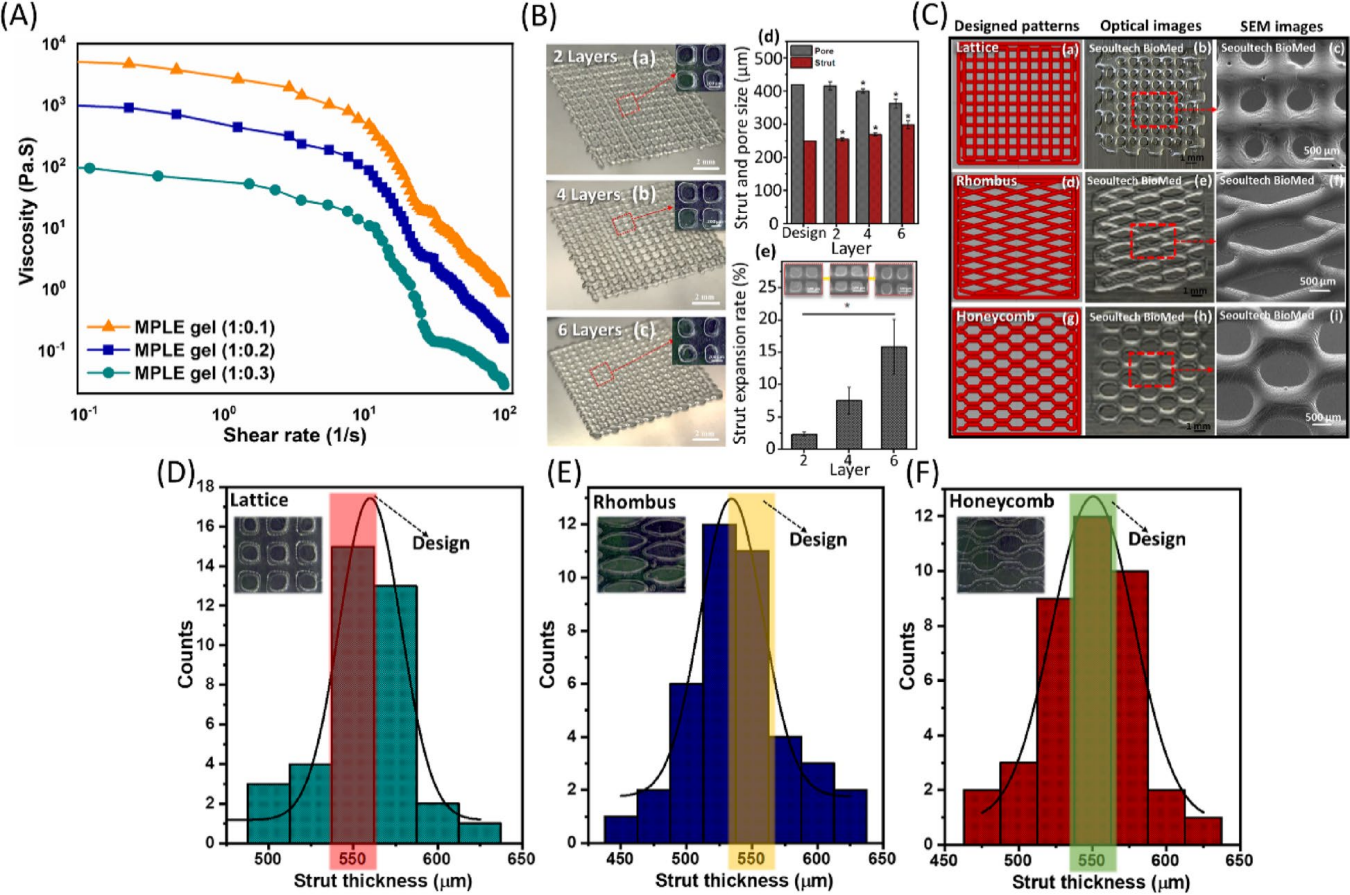
**A** Rheological analysis of the MPLE gels. **B** Optical images of the 3D printed MPLE gel in the lattice infill structures with 2(a), 4(b) and 6(c) layers, respectively; and their strut and pore diameter of the fabricated MPLE (1:0.3) gel scaffolds (d); strut expansion rate comparing design and printed struts (e). **C** the 3D printed MPLE (1:0.3) gel scaffolds in lattice (a-c), rhombus (d-f) and honeycomb (g-i); and the dimensions of initial 3D model designs (a, d & g) and their optical (b, e & h) and SEM images (c, f & i). Histogram graphs comparing the thickness values of the printed struts with the initial designed values (shaded boxes) such as (**D**) lattice, (D) rhombus and (**E**) honeycomb infill structures, where inside small images were obtained by SEM

Figure 4A shows the rheology analyses of the MPLE gels with different amount of PEGDA (1:0.1, 1:0.2, 1:0.3). At zero shear rate, the viscosity of the three gels was measured as 5440.72, 1059.22, and 101.75 Pa.s for the 1:0.1, 1:0.2 and 1:0.3 MPLE gels, respectively. At room temperature, all the gels showed shear-thinning ability even with low shear rates, which indicating that the gels are suitable for extrusion-based 3D printing. Considering the low stress applied, still the gels showed shear-thinning properties up to 100 Pa.s^− 1^ shear rate.

Figure 4Ba-e shows the optical images of the 3D printed lattice structure scaffolds with 2 to 6 layers and their pore size and strut thickness. These graphs represent the difference between the original designs and the 3D printed strut structures. Initial optimization showed that as the printing layers were increasing, the pore size was decreasing slightly, whereas the strut thickness was increasing accordingly. However, further optimization with different parameters yielded high resolution of the multi-layered scaffolds in this study.

The gels showed promising results of rheology analysis, mechanical properties and texture analysis for their application to the extrusion-based 3D printing, as evident from the viscosity data Fig. 4A. Hence, pneumatic-based extrusion 3D printing with the LP-capped and MPLE gels were tried as stated in the methods section with optimized conditions after experimenting by controlling printing conditions. From the optimized process, the MPLE (1:0.3) gel was printed with excellent printability and continuous extrusion without syringe nozzle blockage. High resolution printing of the lattice (Fig. 4Ca-c), rhombus (Fig. 4Cd-f), and honeycomb (Fig. 4Cg-i) structures in 2, 4, 6 layers was obtained by optimizing air pressure and nozzle diameter size. The optical images showed the 2-layer printed structures of the MPLE (1:0.3) gel (Fig. 4C-b, e, h) and its corresponding high magnified SEM images (Fig. 4C-c, f, i) also show their high resolution-printed structures in lattice, rhombus, and honeycomb. The SEM images of the MPLE (1:0.3) gels did not show any porous structures on their surfaces, and the printed layers were smooth with few wrinkles on some areas, which may be due to the freeze-drying process for the SEM analysis. The printed samples showed stable structures without collapsing or drying out and the UV irradiation helped uniform crosslinking of the gel after printing. However, when comparing the post-printing shape fidelities of the three printed structures (Fig. 4D-F), the honeycomb structures showed the best resolution than the other two designs printed in this study. Even though the honeycomb structures are more intricate than the lattice or rhombus, they showed a resolution of 550.42 ± 2.81 μm in strut sizes even with 2 layers of printing. But in all samples, as the layers increased, the resolution started decreasing slightly. However, the resolution was under control as the UV-crosslinking helped both stabilize the layer slipping and increase the layer-to-layer bonding.

The pore structures of the 3D printed MPLE (1:0.3) gels were examined before and after lyophilization (Table 1). Generally, it is considered that the measured porosity in the 3D printed structures would be different from the theoretical ones of the design model used for the extrusion-based 3D printing. The 3D printed structures showed reduced porosities from 30% to ~ 11% and ~ 20% for the wet and dry porosity, respectively, when compared to that of the 3D model design. These differences in the porosity percentage and pore area are mainly attributed to the water content of the gels. Additionally, in all cases, the porosity in the dry state (freeze-dried) was higher than that in the wet state (maximum swelling) as expected. This result was attributed to higher swelling of the struts of the printed wet hydrogels, but increase in porosity due to the removal of water from the printed samples, leading to 40 to 23% difference in porosity [56].

**Table 1:** Porosity changes of the 3D printed MPLE (1:0.3) gel scaffolds in three different designs

Properties	Designed Porosity (%)	Porosity in wet state (%)	Pore area in wet state (mm^**2**^)	Porosity in dry state (%)	Pore Area in dry state (mm^**2**^)
Printed Structure					
**Lattice**	30	10.91 ± 0.17	13.21 ± 0.20	18.13 ± 0.28	21.76 ± 0.34
**Rhombus**	30	11.29 ± 0.54	13.67 ± 0.65	22.21 ± 1.05	26.66 ± 1.27
**Honeycomb**	30	11.72 ± 0.50	14.19 ± 0.61	23.23 ± 1.01	28.15 ± 1.22

**Fig. 5:**
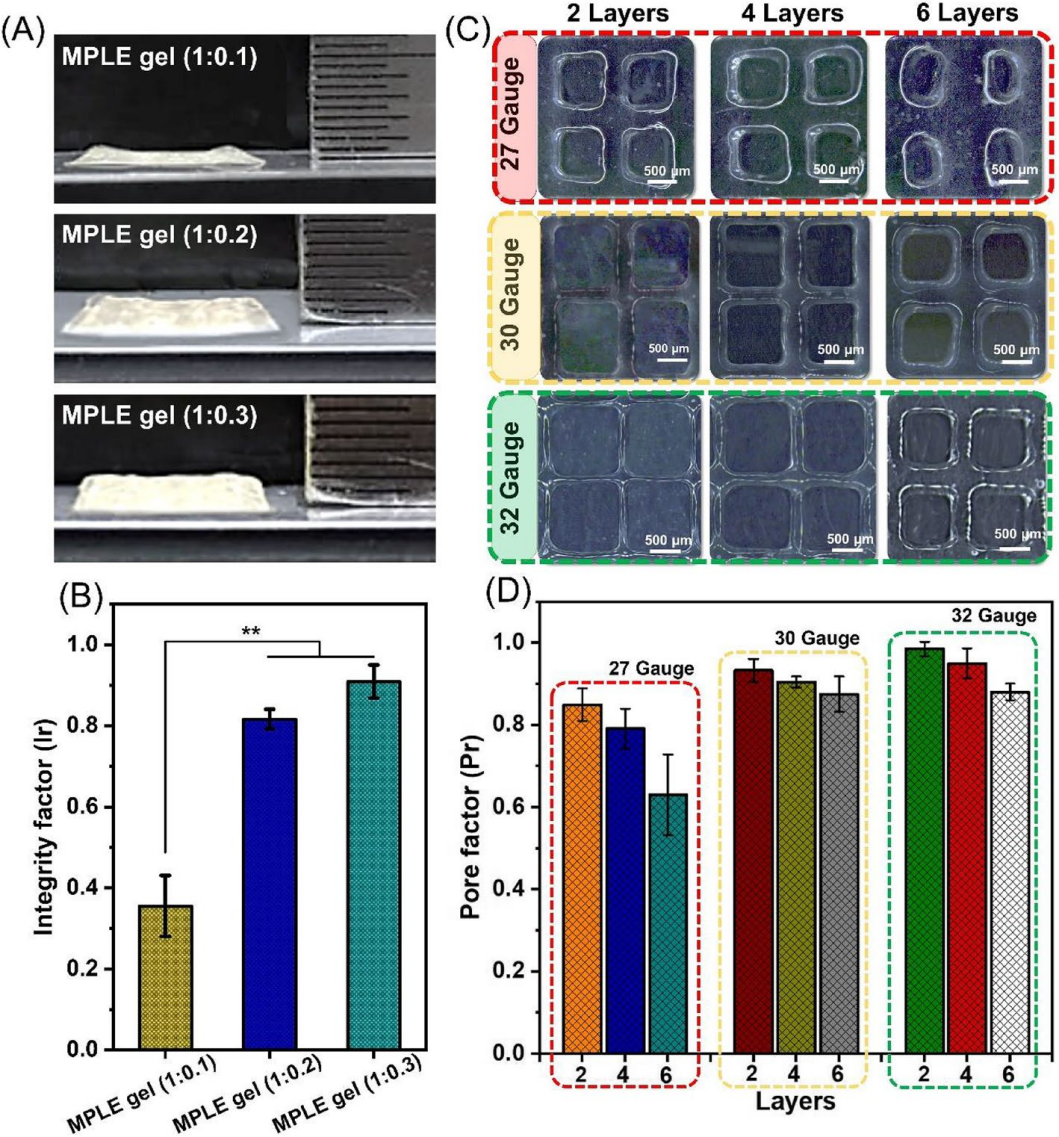
**A** Optical images of the 3D printed scaffolds (10 layers) using the MPLE gels with three ratios of LP-capped gel: PEGDA (1:0.1, 1:0.2, and 1:0.3 w/v). **B** Microscope images of the 3D printed MPLE gel (1:0.3) scaffolds in lattice infill structure with 2, 4, and 6 layers by using different needle sizes (27G, 30G, and 32G). **C** Integrity factor (Ir) of three types of MPLE gels. **D** Pore factor (Pr) of the MPLE gel (1:0.3) scaffolds

To further evaluate shape deviations in vertical z-axis during the printing process, the MPLE gels with varied ratios of LP-capped gel/PEGDA (1:0.1, 1:0.2, and 1:0.3 w/v) were printed up to 10 layers for comparisons (Fig. 5A). the MPLE gel (1:0.3) scaffold could maintain a good structure without collapses. This result was consistent with the integrity (Ir) and pore factor (Pr) measurement (Fig. 5B), where the MPLE gel (1:0.3) scaffold shown the highest Ir value (0.90 ± 0.041) in comparing to only 0.82 ± 0.024 and 0.35 ± 0.075 for the MPLE gel (1:0.2) and MPLE gel (1:0.1) conditions, respectively. The resultant data also indicates that the ratio of LP-capped gel/PEGDA at 1:0.3 was the optimal mixture in this study.

Figure 5C-D represents versatile printability of the MPLE gel (1:0.3) by printing 2, 4 and 6 layers with different nozzle sizes (27, 30 and 32 gauge). It is notable that the Pore factor (Pr) value reached closely to 1.0 (almost square pores) when reducing the size of nozzle to 100 μm (32G) in which the strut thickness and Pr (2-layer sample) were optimized within 90–140 μm and 0.98 ± 0.017, respectively. This result confirms the high-resolution printability of the MPLE gel for their potential applications to complex tissue engineering and regenerative medicine.

### Study on in-vitro cytotoxicity assays and drug release

**Fig. 6:**
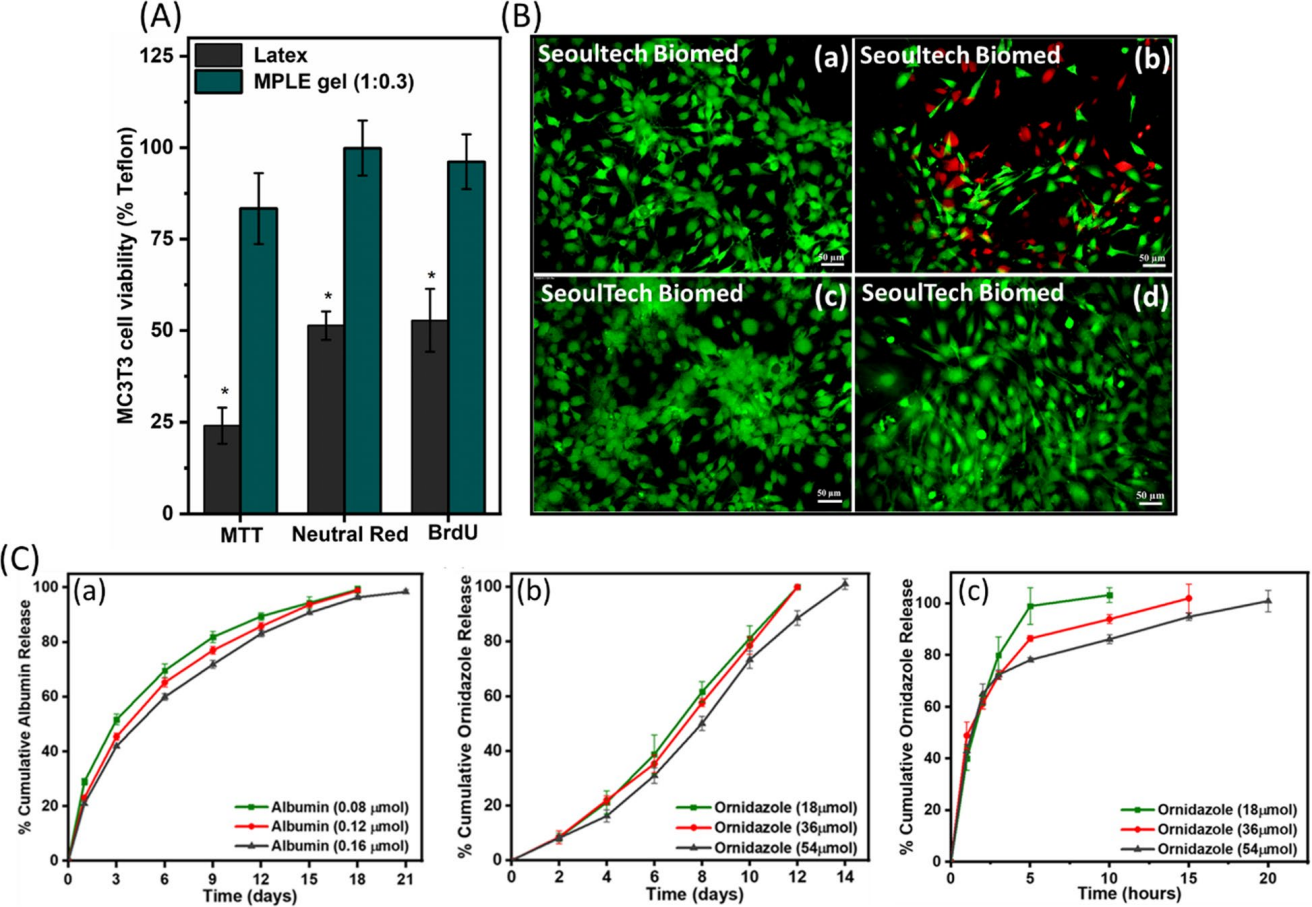
**A** Cytotoxicity assays of the extracts from Teflon, latex, the LP-capped gel, and MPLE gel (1:0.3) samples. **B** Representative Live/Dead (MC3T3-E1 cells) images by the extracts of (a) Teflon (negative control), (b) Latex (positive control), (c) the LP-capped gel, (d) the MPLE gel (1:0.3). **C** In vitro release profiles of a BSA protein and ORN drug from the 3D-printed MPLE gel (1:0.3), where (a) BSA was incorporated inside gel, (b) ORN incorporated inside, (c) adsorbed onto the 3D printed gels. (*p ≤ 0.05)

To test the cytotoxicity of the prepared gels, MTT, Neutral Red and BrdU assays were carried out on the MPLE (1:0.3) gel samples (Fig. 6A). Compared to the positive control, both the MPLE (1:0.3) gel samples showed significantly higher cell viability in all the three assays. While the osteoblast-like cells (MC3T3) were used for this in vitro experiment, the negative and positive controls were Teflon and Latex, respectively. In comparison to the Teflon samples (Fig. 6B-a), the MPLE (1:0.3) gel samples showed excellent cell viability values at 96.13 ± 7.48%, which is an excellent degree of cell viability for its biomaterial applications [57]. Live/dead assay was performed for the MPLE gel (1:0.3) samples till 7 days and compared with the control cell culture plate (not shown) as well as both positive and negative control latex samples (Fig. 6B-a,b). In line with the cytotoxicity assays, the LP-capped and MPLE (1:0.3) gel samples presented high cell friendly environment by allowing many osteoblasts to grow on their surfaces (Fig. 6B-c,d). Compared to the negative control, the gel samples also showed similar degree of live cells with no cell death under the area of observation. The cell numbers increased as the days of incubation increased, and the MPLE (1:0.3) induced cell spreading (Fig. 6B-d). The gels did not have any negative influence on the cells as the phenotype of the cells resembled the control cells without gels or any other external factors. These experiments confirmed that the gels are not toxic to the cells and can be positively considered for the tissue engineering applications.

The ability of the MPLE (1:0.3) gels to incorporate and release other bio-factors like synthetic drugs or proteins on a sustained manner was tested with a model protein and drug (Fig. 6C). Ornidazole (ORN) was taken as the model small molecular weight drug, whereas bovine serum albumin (BSA) was taken as the model long molecular weight protein. Apart from the direct mixing of bioactive factors inside the gel samples, the ORN drug was also adsorbed on to the MPLE gels and its release was studied for comparison. BSA which is a large molecular weight globular protein showed slower release from the MPLE gels (1:0.3) till 21 days. However, in case of the ORN-incorporated gels, the drug release was faster than BSA but slower in contrast to the ORN drug absorbed onto the gels. This faster and slower release is attributed to the molecular weight difference and entanglement of the drug molecules inside the crosslinked MPLE gel networks, which took longer time for its release, respectively. For instance, the adsorbed ORN molecules leached out faster from the surface of the gels within a short time as the drugs were not entrapped inside the cross-linked network. Therefore, the release study results show that the release pattern also can be tuned by using appropriate crosslinking density or method and selecting both delivery modes and appropriate bioactive factors with desirable molecular weights to compensate the release mechanism. The release of ORN drugs is mainly expected to occur in diffusion mechanism due to the hydrolysis that is happening in the crosslinked gel network when swelling. Porous structures present in the gel network allows the bioactive factors to release out faster. However, in case of the proteins, the larger size and higher charges would make effect on their releases. Considering the different properties required for the 3D printed gel scaffolds as explained earlier, bioactive factor incorporation and their release from the gel is also one of the important criteria needed in the extrusion-based 3D bioprinting for their tissue engineering applications.

### In-vivo biocompatibility studies

**Fig. 7:**
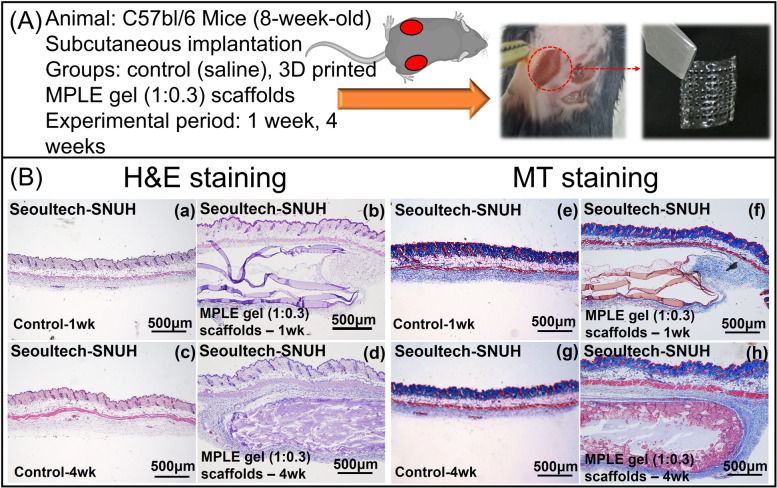
**A** The experimental conditions of the 3D honeycomb-printed MPLE gels (1:0.3) implanted subcutaneously in mice for 1 and 4 weeks. **B** In-vivo histological analysis of (a-d) H&E staining, and (e-h) Masson’s Trichrome staining

After subcutaneously implanting of the 3D printed MPLE gel (1:0.3) samples in mice, the samples were explanted at the time periods of 1 and 4 weeks. The experimental conditions of in-vivo histological analysis were shown in Fig. 7A. H&E staining (Fig. 7B-a,d) showed minor inflammatory responses and degradations were detected after 4-weeks of implantation. In the 3D honeycomb-printed MPLE scaffold (1:0.3) group, hydrogel strands were clearly observed at the 1^st^ week and infiltration of fibroblast-related regenerated tissue was observed mildly at the edge of the implanted-gel strand. Afterward, the surrounding tissue was completely infiltrated along with entire hydrogel strands (Fig. 7B-d). Masson’s trichrome (MT) staining was conducted to examine the distribution of collagen tissue around the implanted sample (Fig. 7Be-h). After 4 weeks of implantation, there was no severe tissue fibrosis (indicated by blue colour) in the 3D printed MPLE gel group. These results show that the MPLE gel (1:0.3) samples were non-toxic and did not elicit any drastic inflammatory effect near the implantation cite or to the animals.

**Fig. 8:**
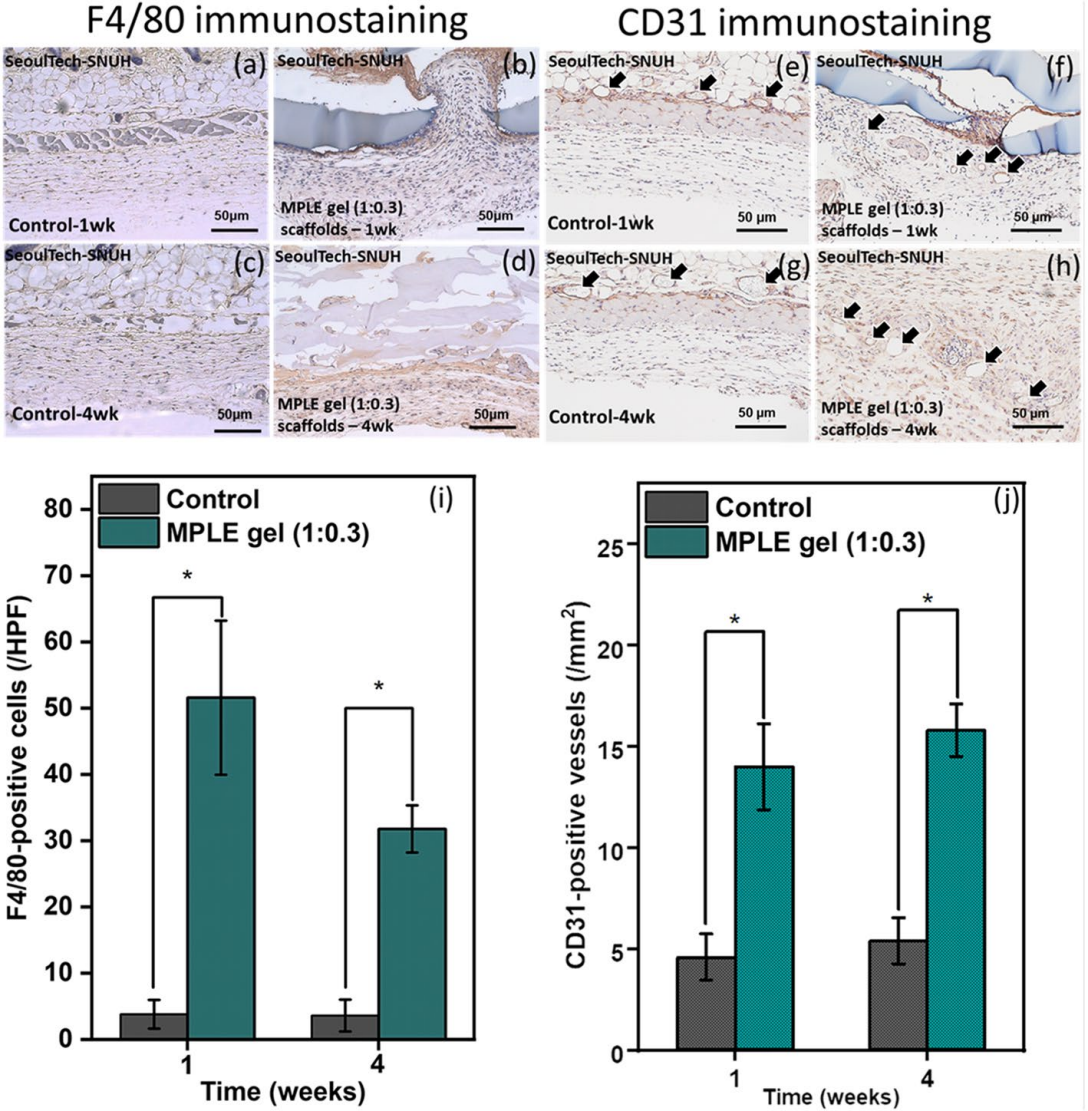
Immunohistochemistry analysis of the 3D honeycomb-printed MPLE gel (1:0.3) samples subcutaneously implanted in mice. Stained with F4/80 (brown color: macrophages) marker, the samples were observed at week 1 (a-b) and 4 (c-d) and CD31 (brown color: blood vessels) marker specified with black arrows as shown at week 1 (e-f) and 4 (g-h). **B** Quantitative values of (i) F4/80 and (j) CD31 immunostaining of the explanted tissues from the saline (control) and 3D printed MPLE gel (1:0.3) samples after subcutaneous implant in mice for 1 and 4 weeks

As shown in Fig. 8, it is evident that the expression of macrophage decreased significantly over time by immuno-histochemical analysis for F4/80 antibody (Fig. 8a-d). The quantitative estimation also showed the reduced macrophages after 4 weeks (Fig. 8i). These results suggest that the implanted 3D honeycomb-printed MPLE gel (1:0.3) are highly biocompatible. Further, in order to examine the ability of forming new blood vessels (angiogenesis), CD31 was used as a blood vessel endothelial marker, (Fig. 8e-h). It is clearly observed that the 3D printed MPLE gel (1:0.3) group showed a statistically significant improvement when comparing to the control sample over time which indicated higher number of blood vessel formation (Fig. 8j). Moreover, the formation of new blood vessels in the 3D printed MPLE gel (1:0.3) group were shown over 1 (Fig. 8g) and 4 weeks (Fig. 8h). In addition, increase in the numbers of blood vessels after implantation suggest that the 3D honeycomb-printed MPLE gel implants has a positive effect in terms of tissue regeneration. The resultant in vivo study implied that the 3D honeycomb-printed MPLE gel is biocompatible and applicable to the biomedical fields such as 3D printing and tissue engineering as well as drug delivery.

## Discussion

Modulation of printable, rheological and mechanical properties by gel components and their molecular weight and compositions is essential for development of an extrusion-based 3D printing hydrogel with high resolution and post-printing shape fidelity by using both natural and synthetic polymers, even though hydrogels with excellent mechanical properties and printability have been reported by several research groups. For instance, Guo et al. reported a mechanically tough hydrogel with double networks synthesized by combining natural polymers of hyaluronic acid and alginate, demonstrating excellent mechanical strength [19]. Even though the double-network hydrogel showed excellent mechanical properties such as toughness, flexibility, adhesiveness with the high cell viability (> 85%), it remains challenging to control molecular weight for high-resolution printability for both in vitro and in vivo studies. A further study by DiCiccio et al. utilized caffeine to catalyze the ring-opening reaction of diglycidyl ether functionalized monomers with citric acid for the synthesis of new tough hydrogel [20]. The caffeine catalyzed gel showed excellent mechanical properties such as toughness, flexibility, adhesiveness with excellent in vitro cytotoxicity against four cell lines (HeLa, HEK293, HT29-MTX-E12, and C2BBe1) and no symptoms of toxicity was found after in vivo oral administration of the gel to rats. However, it was very difficult to control its molecular weight with shear-thinning viscosity for 3D printing in biomedical applications. Therefore, the development of novel printable hydrogel inks with high-resolution printability, excellent mechanical properties and biocompatibility remains necessary.

In this study, we reported the synthesis of new tough hydrogel (LP-capped gel) with excellent self-healing capability (~ 4 h), stretchability (> 20 times its initial length), and toughness (18,206 ± 1966 kJ m^− 3^) and transformed it into printable hydrogel ink (MPLE gel) by adding poly (ethylene glycol) diacrylate via radical polymerization confirmed by the significant decrease of the C=C peak at 1642 cm^-1^ with the degree of conversion at 90.12 ± 4.5% after photos crosslinking 10 min (Supplementary Information). The MPLE gel demonstrated with tunable swell-ability, degradation, post-printing stability and mechanical properties. By altering the LP-capped gel/PEGDA ratios, we found the optimal gel composition to be 1:0.3 (w/v) that enabled the fabrication of 3D printing scaffolds at high resolution (90-140 μm in strut thickness) with various complex geometries (lattice, rhombus, and honeycomb) confirmed by the measurement of the factors of printing integrity (Ir) and pore (Pr). In addition, the long-term release profiles of bioactive molecules were well-controlled by incorporating high molecular BSA (21 days, 98.4 ± 0.69%), or small molecule ORN (14 days, 97.1 ± 1.98%) into the MPLE gel scaffolds for the tests of potential therapeutic applications.

More importantly, the MPLE gels represents excellent in vitro cyto-compatibility against osteoblast-like cells (MC3T3) with viability value at 96.43% ± 7.48% over 7 culturing days. Further advantage of the MPLE gel was demonstrated in in vivo studies, where the flexible gel scaffolds showed significant improvement on angiogenesis with minor inflammatory response after 4-week implantation in mice. These results again confirm the high potential of this printable and adhesive gel for a wide range of applications in the fields of biomedical engineering and tissue regeneration.

## Conclusions

In summary, we successfully developed the 3D printable MPLE hydrogel ink by using both condensation and radical polymerization, i.e. capping the proliferating poly(malate-*co*-propylene oxide) copolymer with mono-functional lipoic acid during condensation reaction processing, and then radical graft-polymerization of the LP-capped gel with chemically inert and biocompatible PEG-DA. The self-healing adhesive LP-capped gel was successfully transformed into a printable gel by controlling the composition of non-adhesive PEGDA during printing process for biomedical applications. The obtained hydrogel showed excellent mechanical properties, 3D printability, in vitro*/*in vivo biocompatibility, drug release with different molecular weights, and adhesiveness as well as control of self-healing by changing its composition. In the future work, we will focus on the effects of different PEGDA concentrations of the MPLE gels on gene and protein expression, as well as both development of simplified sterilization method of the sterilization and optimization of bioink mixing to control the cell viability for bioprinting process.

## Supplementary Information

**Additional file 1: Figure S1.** Step by step fabrication process of MPLE gel and its 3D printed sample in lattice form. **Figure S2.** (A) FTIR spectra of MPLE (1:0.3) gel at different photo-crosslinking times and (B) their C=C bonds degree of conversion. **Figure S3.** Stretchability of MPLE gels at initial stage (A-D) and after extension testing (E-H). **Figure S4.** Photographs of fibers formed by extension of LP-capped gel (A-a, A-b, A-c), and (B) SEM images of the gel fiber at low (in small box) and high magnification. **Figure S5.** In vitro 3D culture of MT3T3 cell on printed MPLE gel scaffolds after 0 (a), 3 (b) and 7 (c)days.

## Data Availability

All data and materials are available upon request.

## Abbreviations

α-MEM: Minimum Essential Medium α
3D: Three dimensions
2D: Two dimensions
BrdU: Bromodeoxyuridine
BSA: Bovine Serum Albumin
FTIR: Fourier-transform infrared spectroscopy
H&E: Hemoxylin and Eosin
IHC: Histology and immunohistochemistry
Ir: Integrity Factor
MA: Maleic acid
MC3T3: Osteoblast precursor cell line
MPLE gel: Poly(maleate-propylene oxide)-lipoate-poly(ethylene oxide)
MT: Masson’s trichrome
MTT: 3-(4,5-dimethylthiazol-2-yl)-2,5-diphenyltetrazolium
LP: Lipoic acid
LP-capped gel: Lipoic acid-capped gel
PBS: Phosphate buffer saline
ORN: Ornidazole
PEGDA: Poly(ethylene glycol) diacrylate
Pr: Pore factor
SEM: Scanning electron microscope
TGA: Thermogravimetric analysis
TPA: Texture profile analysis
UV: Ultraviolet
