# Supplementary material for: Control of maleic acid-propylene diepoxide hydrogel for 3D printing application for flexible tissue engineering scaffold with high resolution by end capping and graft polymerization
Source: Biomater Res. 2022 Dec 9;26:75. doi: 10.1186/s40824-022-00318-x (PMC9733183; doi:10.1186/s40824-022-00318-x)
Supplement: Supplementary file 1 — Additional file 1: Figure S1. Step by step fabrication process of MPLE gel and its 3D printed sample in lattice form. Figure S2. (A) FTIR spectra of MPLE (1:0.3) gel at different photo-crosslinking times and (B) their C=C bonds degree of conversion. Figure S3. Stretchability of MPLE gels at initial stage (A-D) and after extension testing (E-H). Figure S4. Photographs of fibers formed by extension of LP-capped gel (A-a, A-b, A-c), and (B) SEM images of the gel fiber at low (in small box) and high magnification. Figure S5. In vitro 3D culture of MT3T3 cell on printed MPLE gel scaffolds after 0 (a), 3 (b) and 7 (c)days. [file 40824_2022_318_MOESM1_ESM.docx]

**<Supporting Information>**

Control of maleic acid-propylene diepoxide hydrogel for 3D printing application without supporting materials for flexible tissue engineering scaffold with high resolution

Hao Nguyen Tran^1^, Ingul Kim^2^, Jong Heon Kim**^1^**, Eun-Jae Chung^2^, Insup Noh^1,3^

^1^ Department of Chemical and Biomolecular Engineering, Seoul National University of Science and Technology, Seoul 01811, Republic of Korea

^2^Department of Otorhinolaryngology, College of Medicine, Seoul National University Hospital, Seoul 03080, Republic of Korea

^3^ Convergence Institute of Biomedical Engineering and Biomaterials, Seoul National University of Science and Technology, Seoul 01811, Republic of Korea

Correspondence to Insup Noh (insup@seoultech.ac.kr)

(Revised for *Biomaterials Research*, October 27, 2022)

**Methods**

**Stretchability test**: The stretchability of MPLE gel was evaluated by twisting the gel sample (2 mm x 8 mm x 40 mm) in air. Both sides of the samples were manually hold by using tweezers. Afterwards, the upper side was twisted and pulled upward gradually until breakage.

**Gel fiber formation**: The surface morphologies of the LP-capped gel fibres was investigated using scanning electron microscope (SEM; Tescan Vega 3, Czech Republic) with an accelerating voltage of 15 kV. For the gel fibre test, the LP-capped gel was manually stretched into very thin wire/fibres, then plunged in a liquid nitrogen slush for 20 min. Afterwards, the frozen samples were freeze-dried in a lyophilizer at -80 °C for 3 days.

**Degree of conversion (DC) characterized by FTIR spectrometry**: In this test, FTIR was utilized to examine the content of residual of C=C bonds in the MPLE gel (1:0.3) at different photo-crosslinking times (0, 1, 5, 10 min). The degree of conversion (DC) was calculated based on the percentage of the remaining concentration of the aliphatic C=C double bonds (in 1642 cm^−1^) and the highly adsorbed C=O bands (1731 cm^−1^) as a reference peak before and after radical polymerization. All the experiments were performed in triplicate for each sample. The equation (1) was used by adopting previous publications [1-4].

**DC (%) = (1 -** $\frac{\boldsymbol{R}_{\boldsymbol{cured}}}{\boldsymbol{R}_{\boldsymbol{uncured}}}$**) x 100 (%) Eq. (1)**

Where R_cured_ is the absorbance ratio of polymerized aliphatic C=C double bonds and highly adsorbed C=O bands (C=O _polymer_) after exposure to the UV light, while R _uncured_ is the absorbance ratio of unpolymerized aliphatic C=C double bonds and highly adsorbed C=O bands (C=O _unpolymer_) before exposure to the UV light.

**Photo-rheology measurement:** The photo-rheological study was conducted at room temperature (25°C) in parallel-plate mode with 25 mm diameter and 1 mm gap under the presence of UV light exposure. The unpolymerized inks were prepared in different ratios of LP-capped gel and PEGDA (1:0.1, 1:0.2, and 1:0.3 w/v) along with Irgacure 2959 (3% w/w) to obtain the photocurable inks, which were examined by shear rate of γ’ = 0.1 s^-1^. The irradiation started after 120 s and all samples were exposed to UV-light (365 nm, 30 mW/cm2) for a total of 480 seconds.

**Results and discussion**

Fig. S1 showed the sequential steps of MPLE gel preparation at elevated temperature by using digitalized heater, and its 3D printed sample in lattice form.


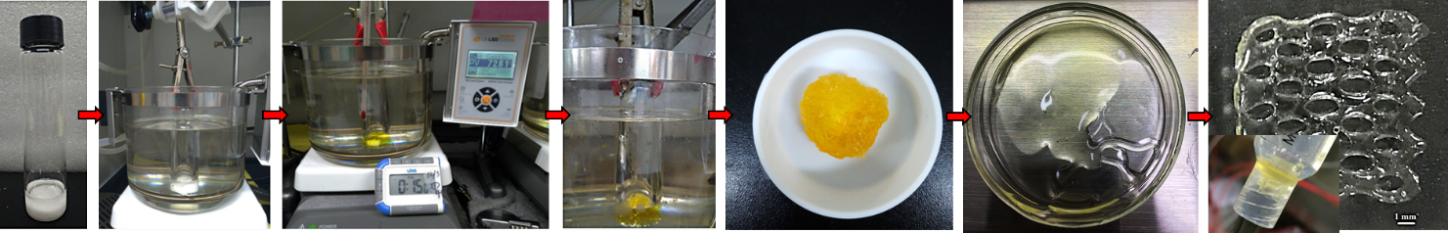


**Fig. S1:** Step by step fabrication process of MPLE gel and its 3D printed sample in lattice form.

MPLE hydrogel were observed by FTIR for their chemical reactions. As shown in the Fig. S2-(A), the significant decrease of the C=C peak at 1642 cm^-1^ (yellow band) referring to C(=O) peaking was clearly observed over time (1, 5, and 10 min) which confirmed the graft polymerization of PEGDA to the LP-capped copolymers under UV exposure. This result was consistent with the data presented in the Fig. S2-(B), where the degrees of conversions at 1, 5 and 10 min were 23.47 ± 0.8%, 48.56 ± 5.4%, and 90.12 ± 4.5%, respectively.


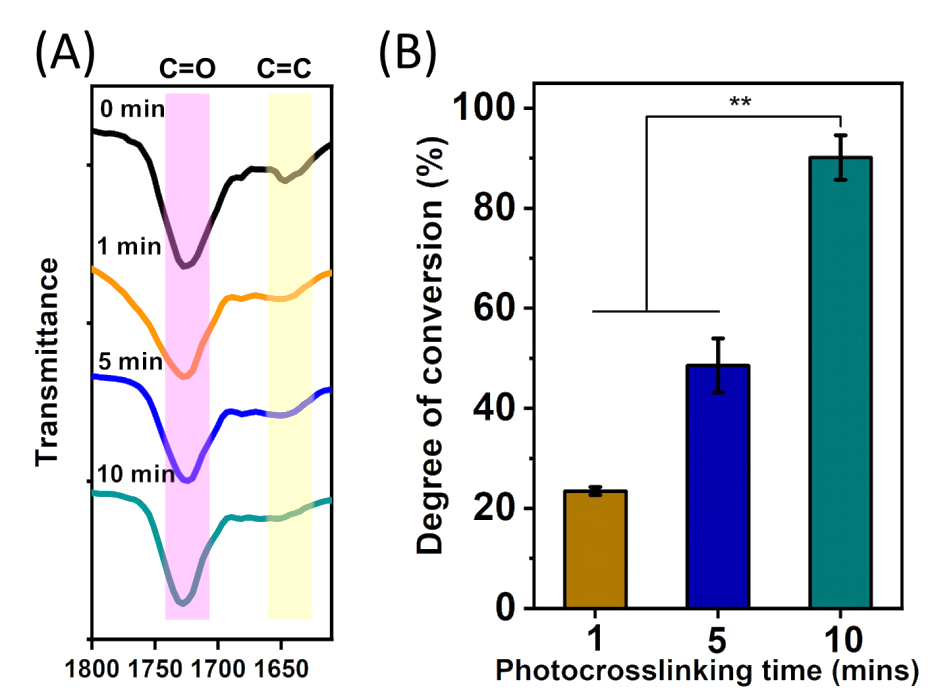


Fig. S2. (A) FTIR spectra of MPLE (1:0.3) gel at different photo-crosslinking times and (B) their C=C bonds degree of conversion.

Stretchabilities of MPLE gels with different ratios of MP:LP, MPL:PEGDA were observed with digital images by holding sample ends with forceps and then twisting them with forceps (Fig. S3). Both MPLE gels and LP-capped gel exhibited good flexible properties at early stage; however, the MPLE gels showed an obvious breakage when increasing the stretching force on the samples which indicates their limited stretchability when comparing to LP-capped gel. This is due to the shrinkage on the surface during the freeze-drying process. Furthermore, the outstanding elongation properties from LP-capped gel can be attributed to the combination between the hydrogen bonding and strong ionic interactions in the 3D cross-linked network.


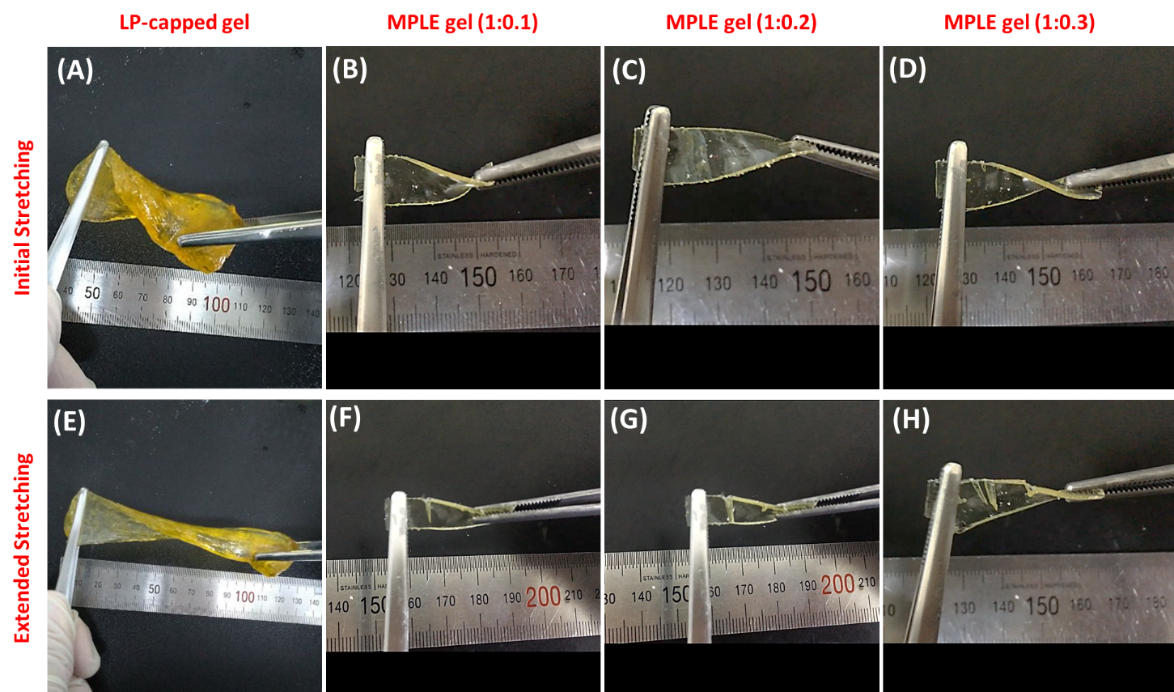


**Fig. S3:** Stretchability of MPLE gels at initial stage (A-D) and after extension testing (E-H).

The fibres in digital images represent fiber formulation from LP-capped gel (Fig. S4A.a-c) where the fiber can be obtained by stretching manually. Fig. 4B reveals the fiber thicknesses ranging from 100-150 µm with rough surface by SEM.


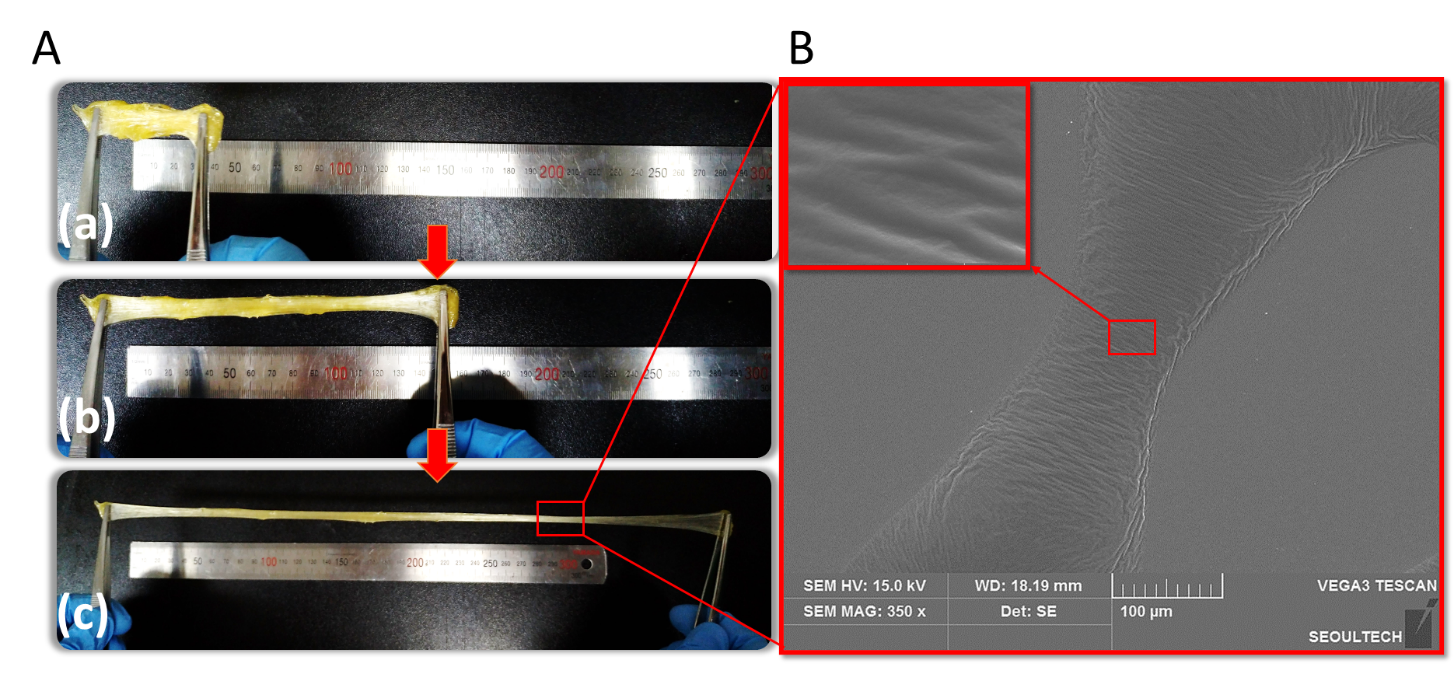


**Fig. S4:** Photographs of fibers formed by extension of LP-capped gel (A-a, A-b, A-c), and (B) SEM images of the gel fiber at low (in small box) and high magnification.

Cell behaviors on the printed gel samples were observed with fluorescence microscopy. As shown in Fig. S5, the MPLE(1:0.3) gel scaffolds showed the gradual increase in terms *of in vitro* cell density and behaviors to 7 days compared to that on day 0, demonstrating its excellent biocompatibility, i.e. cell adhesion and proliferation, for its potential biomedical engineering applications.


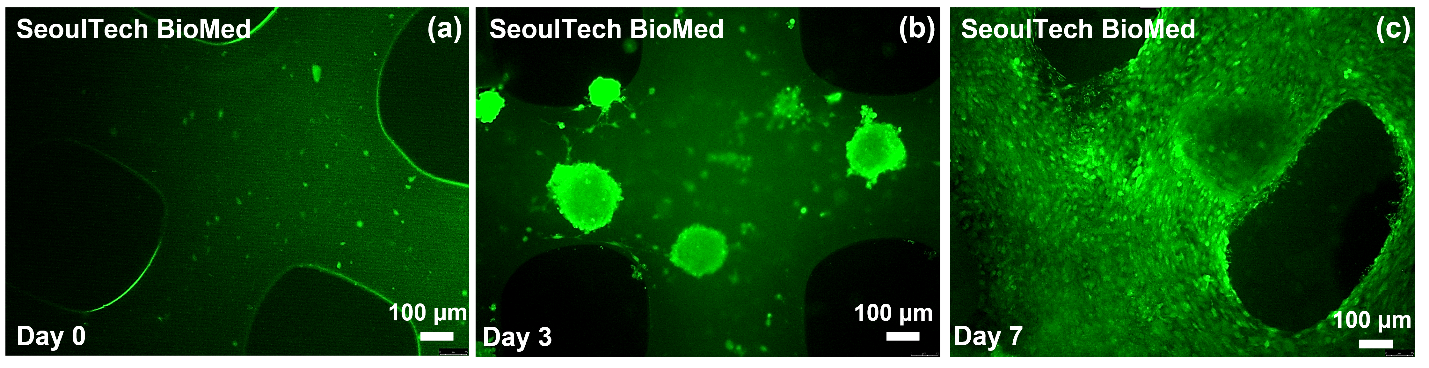


Fig. S5. *In vitro* 3D culture of MT3T3 cell on printed MPLE gel scaffolds after 0 (a), 3 (b) and 7 (c)days.

**3. References**

[1]. Chen, H., Lee, S. Y., & Lin, Y. M. (2020). Synthesis and formulation of PCL-based urethane acrylates for DLP 3D printers*. Polymers*, 12(7), 1500.

[2]. Askari, F., Zandi, M., Shokrolahi, P., Tabatabaei, M. H., & Hajirasoliha, E. (2019). Reduction in protein absorption on ophthalmic lenses by PEGDA bulk modification of silicone acrylate-based formulation. *Progress in Biomaterials*, *8*(3), 169-183.

[3]. Ashjari, H. R., Ahmadi, A., & Dorraji, M. S. S. (2018). Synthesis and employment of PEGDA for fabrication of superhydrophilic PVDF/PEGDA electrospun nanofibrous membranes by in-situ visible photopolymerization. *Korean Journal of Chemical Engineering*, *35*(1), 289-297.

[4]. Belqat, M., Wu, X., Gomez, L. P. C., Malval, J. P., Dominici, S., Leuschel, B., ... & Mougin, K. (2021). Tuning nanomechanical properties of microstructures made by 3D direct laser writing. *Additive Manufacturing*, 47, 102232.
